# TphPMF: A microbiome data imputation method using hierarchical Bayesian Probabilistic Matrix Factorization

**DOI:** 10.1371/journal.pcbi.1012858

**Published:** 2025-03-11

**Authors:** Xinyu Han, Kai Song

**Affiliations:** School of Mathematics and Statistics, Qingdao University, Qingdao, China; Emory University Department of Biology, UNITED STATES OF AMERICA

## Abstract

In microbiome research, data sparsity represents a prevalent and formidable challenge. Sparse data not only compromises the accuracy of statistical analyses but also conceals critical biological relationships, thereby undermining the reliability of the conclusions. To tackle this issue, we introduce a machine learning approach for microbiome data imputation, termed TphPMF. This technique leverages Probabilistic Matrix Factorization, incorporating phylogenetic relationships among microorganisms to establish Bayesian prior distributions. These priors facilitate posterior predictions of potential non-biological zeros. We demonstrate that TphPMF outperforms existing microbiome data imputation methods in accurately recovering missing taxon abundances. Furthermore, TphPMF enhances the efficacy of certain differential abundance analysis methods in detecting differentially abundant (DA) taxa, particularly showing advantages when used in conjunction with DESeq2-phyloseq. Additionally, TphPMF significantly improves the precision of cross-predicting disease conditions in microbiome datasets pertaining to type 2 diabetes and colorectal cancer.

## Author summary

Data sparsity is a significant challenge in microbiome research, as it compromises the accuracy of analyses and obscures important biological relationships. To address this issue, we developed a novel machine learning method called TphPMF, which stands for Phylogenetic Probabilistic Matrix Factorization. This method improves data imputation by incorporating phylogenetic relationships among microorganisms into a probabilistic matrix factorization framework, allowing for more accurate predictions of missing data. Our results demonstrate that TphPMF significantly outperforms existing techniques in recovering missing taxon abundances. Additionally, it enhances the detection of differentially abundant taxa, particularly when used in conjunction with DESeq2-phyloseq, a common differential abundance analysis tool. Moreover, TphPMF substantially improves the accuracy of predicting disease conditions in microbiome datasets related to type 2 diabetes and colorectal cancer. By effectively addressing data sparsity, TphPMF uncovers hidden biological relationships and bolsters the reliability of microbiome analyses. This advancement not only enhances our understanding of microbial communities but also has significant implications for disease prediction and personalized medicine, offering a robust tool for future microbiome research and clinical applications.

## Introduction

The human body is a vast ecosystem, harboring trillions of bacteria and other microorganisms [1]. These microbes are essential for maintaining health and physiological balance, and they also play roles in disease development. With advancements in next-generation sequencing technologies and insights from the Human Microbiome Project (HMP) [1], our understanding of the relationships between human health conditions and microbial communities has significantly deepened [2], generating a vast amount of genomic data. The challenge now lies in effectively extracting valuable information from these data, a crucial aspect of genomic research.

Metagenomic techniques have emerged as powerful tools for exploring the interactions between microbiomes and diseases. Numerous studies have demonstrated a strong link between dysbiosis in the human microbiome and the onset and progression of various diseases. Recent research has highlighted the potential of metagenomic data in clinical and public health applications, aiding in the diagnosis of infectious diseases [3] and establishing correlations with conditions such as Obesity [4], Type 2 Diabetes [5], Colorectal Cancer [6], Inflammatory Bowel Disease [7], Cirrhosis [8], and other tumorous, immune, and metabolic disorders [9]. These findings support the use of metagenomic data as biomarkers for early disease screening, non-invasive diagnosis, and prognosis assessment [3]. However, the high sparsity of metagenomic data, due to technical and economic constraints, remains a challenge. This sparsity is often due to biological zeros—genuine absences of specific species in the sample—or non-biological zeros, which result from limited sequencing depth, sensitivity issues [10], sampling biases, DNA extraction inefficiencies, and PCR amplification preferences [11]. These factors significantly hinder downstream genomic data analysis.

Particularly, the scarcity of microbiome data presents a significant challenge in differential abundance analysis, a critical element in microbiome research aimed at detecting significant compositional differences between groups. To address this, researchers have devised a variety of methods for differential abundance analysis. These include the zero-inflated negative binomial generalized linear model (ZINB-GLM) [12], which offers a more precise estimation of differential abundance; the metagenomeSeq method [13], utilizing a zero-inflated Gaussian mixture model; the DESeq2-phyloseq method [14,15], integrating negative binomial regression with microbiome data structures; the LOCOM method [16], employing a logistic regression model with false discovery rate control; the LinDA method [17], which employs linear models with log-ratio transformations for differential abundance analysis in compositional microbiome data; the ANCOM-BC2 method [18], utilizing multigroup analysis with covariate adjustments and repeated measures; and non-parametric approaches like the Wilcoxon rank-sum test etc, which do not rely on specific distribution assumptions. While these methods all perform statistical tests on sparse data under certain statistical distribution assumptions, the actual data distribution may not necessarily meet these statistical tests, potentially compromising the effectiveness of these statistical methods.

Moreover, data sparsity is also a concern in single-cell RNA sequencing (scRNA-seq) data. To mitigate the challenges posed by data sparsity in genomic analysis, several methods have been developed for recovering gene expression from scRNA-seq data, including scImpute [19], MAGIC [20], SAVER [21], ALRA [22] and deep learning-based methods such as DeepImpute [23] and DCA [24]. However, these methods often neglect the valuable phylogenetic information of taxa in microbiome data analysis [25–31] and struggle to balance the biological significance of missing data, imputation accuracy, and processing speed. In 2021, Ruochen Jiang et al. introduced a novel estimation method for microbiome data—mbImpute [32]—which enhances data imputation by leveraging information from similar samples, taxa, and optional metadata, including sample covariates and taxonomic phylogenetic signals. In 2022, Yanyan Zeng et al. introduced mbDenoise [33], a method for improving microbiome data analysis by addressing zero-inflation and sparsity. Using a zero-inflated probabilistic principal components analysis (ZIPPCA) model, mbDenoise distinguishes between true zeros and spurious ones, reducing noise and enhancing the representation of microbial community structure. Nonetheless, these two methods do not fully account for the taxonomic phylogenetic relationships between species.

In this paper, we introduce a new microbiome data imputation model based on the Taxa-phylogenetic-based Probabilistic Matrix Factorization method—TphPMF. Originally used in recommendation systems, Probabilistic Matrix Factorization predicts a user’s preferences based on others’ ratings [34]. By incorporating phylogenetic signals and structural matrix information as prior knowledge, TphPMF has demonstrated superior performance in addressing data sparsity in microbiome datasets compared to other methods.

## Results

### TphPMF recovers missing taxonomic abundances more effectively

**Fig 1:**
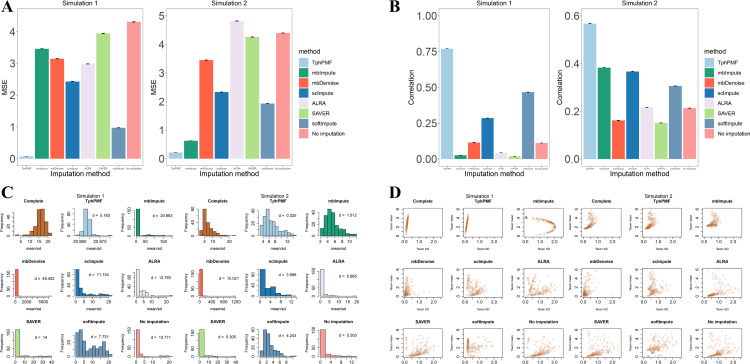
Comparison of the performance of TphPMF with other imputation methods in Simulation 1 and Simulation 2. **A.** Mean squared error (MSE) between imputed and complete data (Performed 100 iterations). **B.** The mean Pearson correlation between imputed and complete data across all taxa (Performed 100 iterations). **C.** Distribution of taxa abundance mean/sd in imputed and complete data, along with the Wasserstein distance between them. **D.** Relationship between the mean and sd of taxa abundance in imputed data and complete data.

To assess the effectiveness of TphPMF in recovering missing taxonomic abundances from microbiome datasets, we conducted three simulation studies, detailed in S1 File. These studies compared TphPMF against three established scRNA-seq imputation methods—scImpute [19], SAVER [21], and ALRA [22], and two microbiome data imputation methods—mbImpute [32] and mbDenoise [33], and one general imputation method, softImpute [35]. All simulation studies were based on the whole-genome sequencing (WGS) dataset of Type 2 Diabetes (T2D) collected by Karlsson et al. in 2013 [36]. To obtain a “complete” taxonomic count matrix without zeros, and thus compare the imputed data with the complete data to evaluate the performance of the imputation methods, we fitted three models based on the real dataset to generate complete data: in simulation 1, a probabilistic matrix factorization (PMF) model was used; in simulation 2, which is similar to Jiang et al. 2021 [32], a linear model that leverages similarities among samples and taxonomic groups in the count matrix was used:


$${\text{Y}}_{\text{ij}}={\text{Y}}_{\text{i}\cdot }^{\text{T}}{\text{α}}_{\text{j}}+{\text{Y}}_{\cdot \text{j}}^{\text{T}}{\text{β}}_{\text{i}}+\epsilon_{\text{ij}}$$

where ${\text{Y}}_{\text{i}\cdot }\in {\text{R}}_{>0}^{\text{M}}$ represents the abundance of M taxonomic groups in the i-th sample, ${\text{α}}_{\text{j}}$ represents the weights of M taxonomic groups for predicting the abundance of the j-th taxonomic group (the j-th term being zero); ${\text{Y}}_{\cdot \text{j}}\in {\text{R}}_{>0}^{\text{N}}$ represents the abundance of taxonomic group j across N samples, ${\text{β}}_{\text{i}}$ represents the weights for N samples when predicting sample i (the i-th term being zero); $\epsilon_{\text{ij}}$ is the error term. In simulation 3, a semi-simulation approach was employed, where missing values in the abundance matrix were replaced with normally distributed random variables, using the observed non-zero values to generate a complete dataset. To introduce more realistic non-biological zeros into the complete data, we emulated the zero-pattern observed in the real dataset, combined with the mixture model we used to identify non-biological zeros to generate the required zero-inflated data. Subsequent application of the seven imputation methods to the zero-inflated data allowed us to evaluate their performance across four metrics: (1) the Mean Squared Error (MSE) between the imputed data and the complete data; (2) the mean Pearson correlation between imputed and complete data across all taxa; (3) the distribution of taxa abundance mean/sd in the imputed data and the complete data, and their Wasserstein distance; (4) the relationship between the mean and sd of taxa abundance in the imputed data and the complete data. We present the results of these imputation methods in Fig 1 (for simulation 1 and 2) and S1 Fig (for simulation 3). Fig 1A and 1B illustrate that TphPMF achieved the lowest MSE and highest Pearson correlation, respectively, indicating superior imputation accuracy. Fig 1C shows that the taxonomic group abundances imputed by TphPMF exhibited the smallest Wasserstein distance to the true distributions, suggesting minimal disparity between the imputed and actual abundance distributions. Fig 1D highlights that the distribution characteristics of the data imputed by TphPMF closely align with those of the complete dataset, underscoring TphPMF’s superiority in restoring distribution characteristics. The results of simulated 3 also demonstrate the effectiveness of our method, TphPMF (S1 Fig). In the simulation, we generated zero inflated data through a binomial distribution. Therefore, by adjusting the parameters in the binomial distribution, we can obtain simulation data with different zero proportions (from 60% to approaching 80%). Overall, TphPMF demonstrated the most optimal performance among the tested imputation methods under different zero proportions (S2-S4 Figs). In addition, we conducted a comparison of the running times (averaged over ten runs) of three microbiome data imputation methods—TphPMF, mbImpute, and mbDenoise—across three simulations. The results (S5 Fig) revealed a significant difference in computational efficiency among the methods. Specifically, TphPMF demonstrated exceptional speed, completing imputation on the entire dataset in less than one second. In contrast, both mbImpute and mbDenoise required considerably more time, with execution times exceeding ten seconds for each imputation. These findings suggest that TphPMF may be a more computationally efficient choice for large-scale imputation tasks, especially when rapid processing is critical.

### TphPMF exhibits superior performance in enhancing the accuracy of DA taxa identification

**Fig 2:**
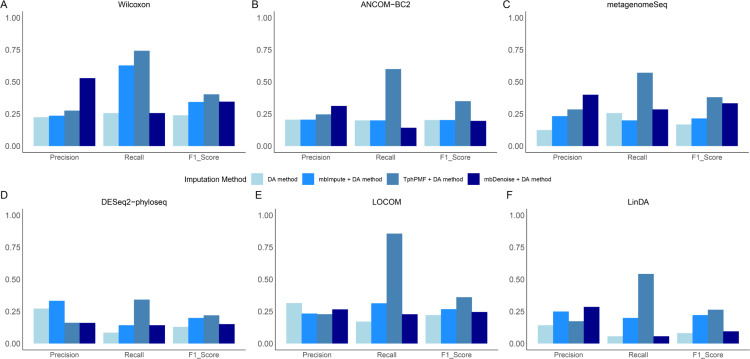
Precision, recall, and F1 score of six DA methods with and without imputation using mbImpute, mbDenoise or TphPMF. **A.** Wilcoxon rank-sum test. **B.** ANCOM-BC2. **C.** metagenomeSeq. **D.** DESeq2-phyloseq. **E.** LOCOM. **F.** LinDA.

Differential abundance (DA) analysis plays a critical role in microbiome research by identifying taxa with significant changes in relative abundance across different conditions. This analysis is essential for understanding microbiome dynamics, microbe-host interactions, disease mechanisms, and ecosystem functions. To further assess the imputation performance of TphPMF on 16S rRNA sequencing data, we utilized the 16S data simulator, sparseDOSSA, to generate “true data” consisting of abundances for 150 taxa, including 35 pre-defined true DA taxa, across 100 samples under two conditions [32]. We subsequently applied TphPMF to impute this sparse dataset to obtain the estimated data. The accuracy of DA taxa identification was assessed using six advanced methods: the Wilcoxon rank-sum test, ANCOM-BC2 [18], metagenomeSeq [13], DESeq2-phyloseq [14,15], LOCOM [16] and LinDA [17], applied to both the original and the imputed datasets. The evaluation focused on three metrics: (1) Precision; (2) Recall; (3) F1 score. As illustrated in Fig 2, all differential abundance analysis methods exhibited better overall accuracy in identifying DA taxa from datasets estimated by TphPMF. Additionally, we tested the imputation method mbImpute [32] and mbDenoise [33], specifically designed for microbiome data, on the original data and applied the same six DA analysis methods to the imputed data. The results showed that while mbImpute and mbDenoise also enhanced the accuracy of DA taxa identification, TphPMF exhibited superior overall performance in improving DA taxa identification accuracy. In addition, we also test different number of pre-defined true DA taxa (20 for S6 Fig and 45 for S7 Fig). These results suggest that TphPMF can more effectively handle sparse microbiome data, thereby enhancing the reliability and accuracy of differential abundance analysis, which may help reveal more potential biological mechanisms and microbe-host interactions, further advancing disease diagnosis and treatment, ecosystem management, and microbial engineering.

### Robustness analysis of TphPMF

**Fig 3:**
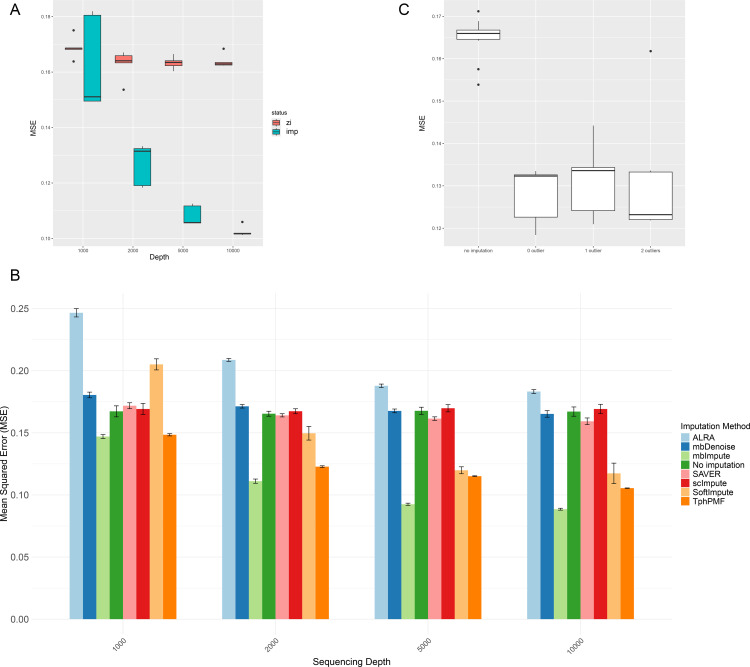
Robustness analysis results for TphPMF. **A.** The MSE of TphPMF at four different sequencing depths (1000, 2000, 5000, and 10000 reads per sample respectively). Here, “zi” denotes zero-inflated, “imp” denotes imputed data. **B.** Comparison of the estimation accuracy (MSE) of different imputation methods at four different sequencing depths. **C.** The estimation accuracy (MSE) of data without imputation, with imputation by TphPMF, and introduced one or two outlier samples after imputation.

We evaluated the robustness of TphPMF from two perspectives: sequencing depth and outlier samples [32]. To achieve this, we simulated complete abundance data for 54 samples and 300 taxa based on the 16S rRNA sequencing data of 54 healthy human fecal samples from the R package HMP16SData [37]. Following data preprocessing, we adjusted the dataset to four different sequencing depths (1000, 2000, 5000, and 10000 reads per sample) to generate complete data at varying sequencing depths. Subsequently, we employed the same nonparametric process as in the previous simulation studies to generate zero-inflated data at each sequencing depth. After applying TphPMF to impute the zero-inflated data, we analyzed the estimation accuracy of TphPMF across different sequencing depths. Fig 3A illustrates a decrease in the mean squared error of TphPMF estimates as sequencing depth increases, indicating enhanced accuracy—an expected outcome since higher sequencing depths reduce the proportion of missing data, thereby providing a richer dataset for model training. Furthermore, we evaluated other imputation methods under varying sequencing depths; Fig 3B shows that methods like ALRA, mbDenoise, and softImpute also exhibit improved accuracy as sequencing depth increases, with TphPMF generally outperforming these alternatives, but not better than mbImpute. To introduce outlier samples, we assigned the highest abundance values to the low-abundance taxa in existing samples and set the abundances of other taxa to zero. A similar process was used to generate more outlier samples. Fig 3C indicates that TphPMF maintains robust MSE performance in the presence of outliers. What’s more, we verified TphPMF’s resistance to the influence of outlier samples by selecting the abundance distributions of four example taxa. S8 Fig confirms that the presence of outlier samples does not distort the distributions of estimated non-outlier samples.

### TphPMF enhances performance of DESeq2-phyloseq

The utility of TphPMF was further demonstrated through its performance on real datasets. By conducting differential abundance analyses on two type 2 diabetes (T2D) whole-genome sequencing (WGS) datasets [36,38] and four colorectal cancer (CRC) WGS datasets [39–42], and then performing a deeper analysis and comparison of the results of DESeq2-phyloseq, we found that TphPMF offers advantages when used in conjunction with DESeq2-phyloseq [32].

Initially, we employed six differential abundance analysis methods, as previously mentioned, to identify differentially abundant (DA) taxa between disease and control samples. Table 1 displays that, at a false discovery rate (FDR) threshold of 0.05, only methods including ANCOM-BC2, DESeq2-phyloseq, and LOCOM successfully identified DA taxa across all of the real raw datasets and the corresponding datasets imputed by TphPMF. To further validate the accuracy of the identified DA taxa, we examined the *P* value distributions of taxa from ANCOM-BC2, DESeq2-phyloseq, and LOCOM across all raw and imputed datasets. S9 Fig indicates that *P* value distributions from DESeq2-phyloseq align with expected results, where the *P* value distributions of taxa should be close to 0 overall, with a uniform trend in the non-zero portion of *P* values. However, the *P* value distributions from ANCOM-BC2 and LOCOM exhibited anomalies. Therefore, we decided to focus our analysis on the efficacy of combining TphPMF with DESeq2-phyloseq.

**Table 1:** The number of differentially abundant (DA) taxa identified in the raw datasets and the TphPMF-imputed datasets of two T2D datasets and four CRC datasets using six DA analysis methods, under an FDR threshold of 0.05.

		Wilcoxon	ANCOM-BC2	MetagenomeSeq
	Total taxa	Raw/Imputed	Raw/Imputed	Raw/Imputed
***Qin et al.* [** **38** **]**	179	29/95	37/102	46/12
***Karlsson et al.* [** **36** **]**	181	8/8	54/60	13/1
***Feng et al.* [** **39** **]**	216	26/54	44/81	33/1
***Vogtmann et al.* [** **40** **]**	210	0/0	37/84	10/0
***Yu et al.* [** **41** **]**	199	11/81	40/94	40/2
***Zeller et al.* [** **42** **]**	237	9/147	38/110	28/5
		**DESeq2_phyloseq**	**LOCOM**	**LinDA**
		**Raw/Imputed**	**Raw/Imputed**	**Raw/Imputed**
***Qin et al.* [** **38** **]**		80/116	36/93	28/107
***Karlsson et al.* [** **36** **]**		53/60	24/46	3/38
***Feng et al.* [** **39** **]**		95/106	31/79	25/94
***Vogtmann et al.* [** **40** **]**		60/50	17/18	0/6
***Yu et al.* [** **41** **]**		77/112	38/81	15/89
***Zeller et al.* [** **42** **]**		104/135	46/114	8/117

**Fig 4:**
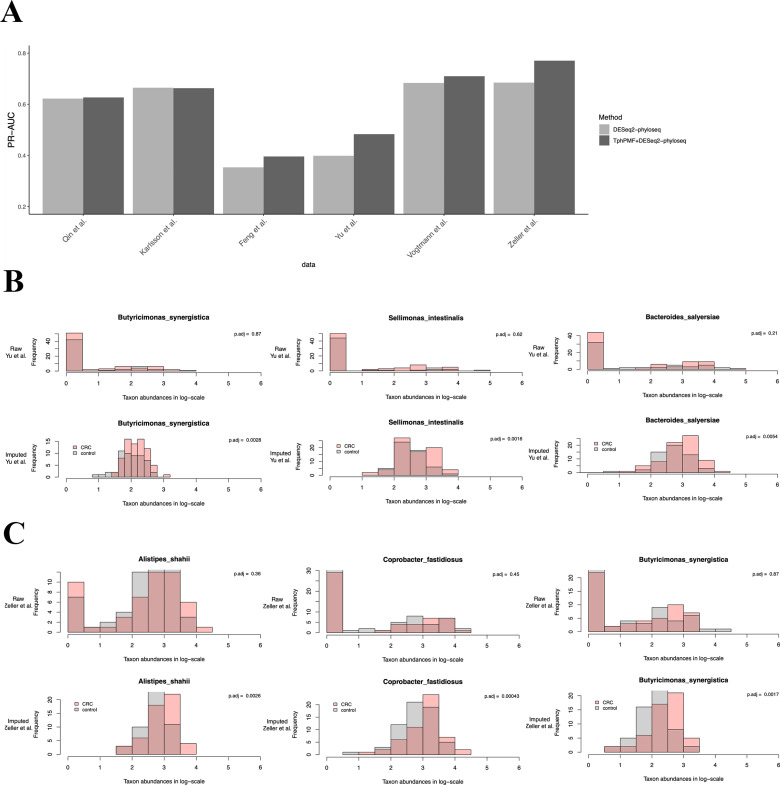
Results of sample disease state prediction using SVM algorithm with DA taxa identified by DESeq2-phyloseq as features, before and after imputation by TphPMF. **A.** PR-AUC for disease state classification prediction. **B.** Abundance distribution of three example taxa identified by DESeq2-phyloseq after TphPMF, which were not identified before imputation, in the dataset by Yu et al. [41], where the upper three graphs represent the abundance distribution before imputation, and the lower three graphs represent the distribution after imputation. **C.** Abundance distribution of three example taxa identified by DESeq2-phyloseq after TphPMF, which were not identified before imputation, in the dataset by Zeller et al. [42].

Correctly identified DA taxa may serve as meaningful disease biomarkers for early detection or treatment. We used the DA taxa identified by DESeq2-phyloseq in both raw and TphPMF-imputed datasets as features to predict disease states using the Support Vector Machine (SVM) and XGBoost algorithm and evaluated the predictions with the area under the precision-recall curve (PR-AUC) from 5-fold cross-validation. The result (Figs 4A and S10) suggests that overall, TphPMF enhances the ability of DESeq2-phyloseq to identify a greater number of accurate DA taxa. We also found that the inclusion of TphPMF most significantly improved the accuracy of disease prediction for the datasets from Yu et al. [41] and Zeller et al. [42] from the results of the SVM algorithm, prompting us to analyze the results from these two datasets more deeply. On examining the DA taxa detected by DESeq2-phyloseq in these two original CRC datasets and their corresponding imputed datasets, we found there are both unique and non-overlapping DA taxa within the original and imputed datasets. Based on these findings, we selected three taxa from those detected as DA post-estimation for further investigation of their abundance distribution for these two CRC datasets, respectively. Literature evidence supported that these taxas, *Butyricimonas synergistica* [43], *Sellimonas intestinalis* [44]*, Bacteroides salyersiae* [45], and *Coprobacter fastidiosus* [46], were reported to be associated with CRC disease. From Fig 4B and 4C, we observed for each microbe that the non-zero abundance range in both the raw and imputed data were similar, and both indicated that the taxa were more abundant in disease samples. However, the prevalent zero distribution in the raw sample data obscured the abundance differences. Similarly, we studied three example taxa from those captured before imputation and not captured after, inspecting their abundance distribution. S11 Fig did not show a clear enrichment of each microbe in disease samples, but there was a significant difference in the zero proportion between disease and control samples. If this was the reason that DESeq2-phyloseq classified them as DA taxa, then we have grounds to believe these taxa may not represent true DA taxa.

Finally, we analyzed the overlap of DA taxa identified by six DA analysis methods across the original T2D datasets, CRC datasets, and their corresponding imputed datasets. Table 2 demonstrates that TphPMF, overall, assists most DA analysis methods in capturing more overlapping DA taxa between different datasets of the same disease type, signifying its importance in enhancing the efficacy of DA analysis methods. Of the twenty DA taxas identified for T2D by ANCOM-BC2 using only TphPMF imputed data sets, *Bacteroides fragilis (BF)* increased significantly in patients who took pravastatin or type 2 diabetes (T2D) mice treated with pravastatin [47,48]. *Bifidobacterium bifidum* showed a potential for decreasing FBG concentration and alleviating insulin resistance [49,50]. Recent studies show that *Flavonifractor plauti*i was significalty associated with T2D [51,52]. *Ruminococcus gnavus* was also reported to be associated with T2D in previous study [53]. For CRC, *Bacteroides fragilis* was reported to be as a potential prognostic factor in colorectal cancer [54]. The abundance of *Roseburia inulinivorans* were reported to be signicantly lower in the CRC subjects than normal subjects [55].

**Table 2:** Analysis results of the overlap of DA taxa captured by six DA analysis methods in the original and TphPMF-imputed datasets for two T2D and four CRC datasets. The first column shows the number of overlapping DA taxa in the original and TphPMF-imputed datasets for two T2D datasets; the second column shows the number of overlapping DA taxa in the original and TphPMF-imputed datasets for four CRC datasets; the third column shows the proportion of overlapping taxa in the total DA taxa identified in the original and TphPMF-imputed datasets for two T2D datasets; the fourth column shows the proportion of overlapping taxa in the total DA taxa identified in the original and TphPMF-imputed datasets for four CRC datasets.

	Overlap_T2D	Overlap_CRC	DR_T2D	DR_CRC
	Raw/Imputed	Raw/Imputed	Raw/Imputed	Raw/Imputed
**Wilcoxon**	0/0	0/0	0.00/0.00	0.00/0.00
**ANCOM-BC2**	10/30	0/5	0.12/0.22	0.00/0.02
**MetagenomeSeq**	1/0	0/0	0.02/0.00	0.00/0.00
**DESeq2_phyloseq**	11/11	2/3	0.14/0.10	0.01/0.02
**LOCOM**	0/17	0/1	0.00/0.14	0.00/0.01
**LinDA**	0/16	0/1	0.00/0.12	0.00/0.01

### TphPMF preserves the distributional features of taxa non-zero abundances

**Fig 5:**
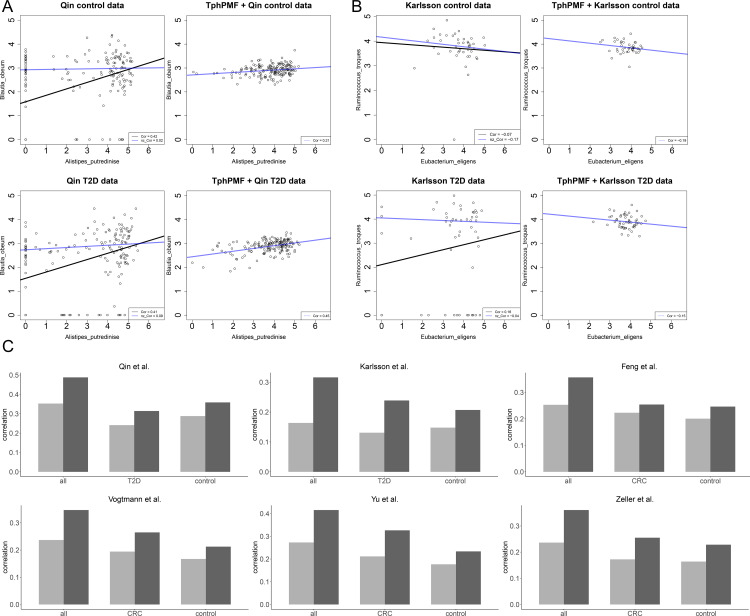
Performance results of TphPMF in preserving the distributional features of taxa non-zero abundances. **A.** Results of the abundance distribution feature analysis for a pair of taxa in the T2D dataset by Qin et al [38]. The upper part of the figure represents the results using contrast samples, while the lower part represents the results using T2D samples. In each part, the left graph shows the results of performing two SMA regressions and calculating Pearson correlation coefficients in the original dataset (using all samples and non-zero abundance samples); the right graph shows the results of performing SMA regression and calculating Pearson correlation coefficients for all samples in the dataset imputed by TphPMF. **B.** Results of the abundance distribution feature analysis for a pair of taxa in the T2D dataset by Karlsson et al [36]. **C.** Comparison of Pearson correlations between the full-sample abundances and the original non-zero sample abundances before and after imputation with TphPMF for all samples, “disease” samples, and control samples across 2 T2D datasets and 4 CRC datasets. The light-colored bars represent the Pearson correlation between the original full-sample and the original non-zero sample abundances, while the dark-colored bars represent the Pearson correlation between the imputed full-sample and the original non-zero sample abundances.

The utility of TphPMF in preserving the distributional features of microbial taxa non-zero abundances has been validated through its integration with differential abundance (DA) analysis methods. To investigate this further, we analyzed the Pearson correlation coefficients of non-zero abundances for a selected pair of taxa from the type 2 diabetes (T2D) whole-genome sequencing (WGS) datasets [32] by Qin et al. [38] and Karlsson et al. [36]. These coefficients were calculated on a logarithmic scale for both raw and TphPMF-imputed abundance data. In the original dataset, correlation coefficients were computed separately using data from all samples and only non-zero abundance samples. Conversely, in the TphPMF-imputed data, where zero abundances are absent, coefficients were calculated across all samples. Fig 5A and 5B illustrate that TphPMF enhances the congruence between the full-sample and the original non-zero sample correlations of paired taxa abundances. Based on these results, we also investigated the linear relationship between paired taxa abundances using Standard Major Axis (SMA) regression. Similar to the analysis mentioned above, in the raw data, two SMA regressions were conducted: one using all samples and another using only non-zero abundance samples. In the TphPMF-imputed data, SMA regression was performed using all samples only. Observations from Fig 5A and 5B reveal significant discrepancies between the regression lines of the original full-sample and non-zero sample, with differences sometimes extending to the direction of the slopes. By contrast, after the imputation by TphPMF, the full-sample regression line closely matches the slope and direction of the original non-zero sample regression line. This result once again confirms that TphPMF excels in preserving the distributional features of taxa non-zero abundances.

Furthermore, we systematically evaluated the performance of TphPMF in the domain of preserving taxa non-zero abundance distributions by comparing the Pearson correlation between the original full-sample abundances, the imputed full-sample abundances, and the original non-zero sample abundances across six real datasets. Fig 5C significantly demonstrates that the inclusion of TphPMF improved the preservation of taxa abundance correlations in both “disease” and “control” groups, as well as across all samples. Additionally, we found that similar results were obtained when using Spearman’s correlation to define similarity (see S12 Fig). These results suggest that TphPMF may have meaningful implications in the realm of recovering inter-taxa correlations.

### TphPMF enhances cross-prediction classification accuracy

**Fig 6:**
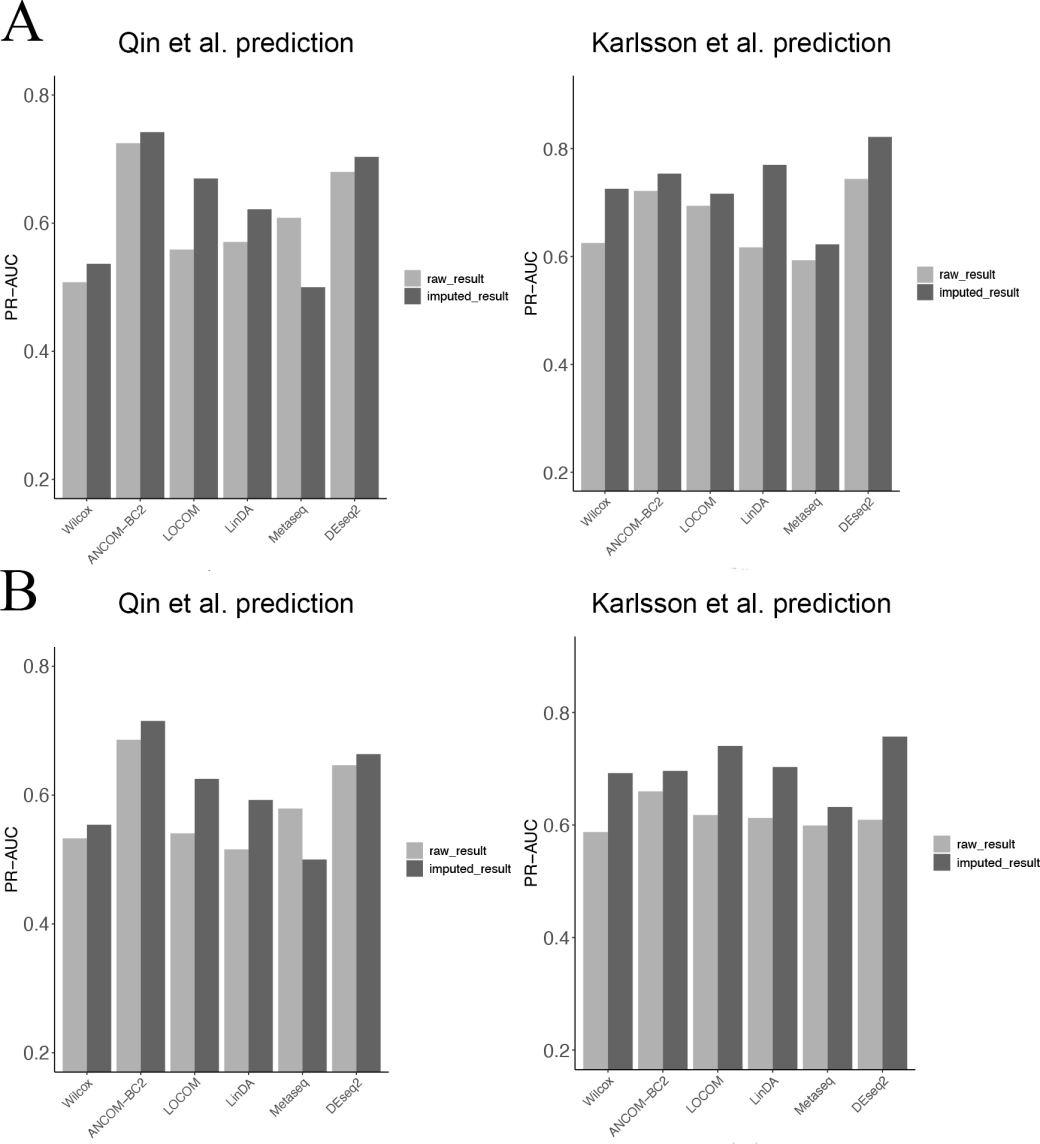
Accuracy of cross-prediction classification (PR-AUC) results using two classification algorithms for the two T2D datasets by Qin et al. [ **38] and Karlsson et al. [36]** The left graph shows the predictive classification results for the Qin et al. [38] dataset using differentially abundant (DA) taxa as features obtained from Karlsson et al. [36]‘s original dataset (light-colored bars) and the dataset imputed by TphPMF (dark-colored bars); the right graph displays the predictive classification results for the Karlsson et al. [36] dataset using DA taxa as features obtained from Qin et al. [36]’s original dataset (light-colored bars) and the dataset imputed by TphPMF (dark-colored bars). **A.** Random Forest. **B.** XGBoost.

Our two cross-experiments further confirmed the outstanding performance of TphPMF in conjunction with DA methods for predicting disease status. For the T2D datasets collected by Qin et al. [38] and Karlsson et al. [36], we attempted to use the differentially abundant (DA) taxa identified through several existing differential abundance analysis methods, including Wilcoxon rank-sum test, ANCOM-BC2 [18], LOCOM [16], LinDA [17], metagenomeSeq [13], and DESeq2-phyloseq [14,15], as features from both the original dataset of Karlsson et al. [36] and the TphPMF-imputed dataset. We then predicted disease status in the Qin et al. [38] dataset using classifiers such as Random Forest, XGBoost, Linear kernel SVM, and Gaussian kernel SVM, with the evaluation of predictive accuracy based on the area under the precision-recall curve (PR-AUC) from 5-fold cross-validation. Similarly, we compared the disease status prediction results for the Karlsson et al. [36] dataset using DA taxa as features obtained from the original and imputed datasets of Qin et al. [38] The results indicate that in most cases, TphPMF generally enhances the accuracy of cross-prediction classification (Figs 6 and S13).

**Fig 7:**
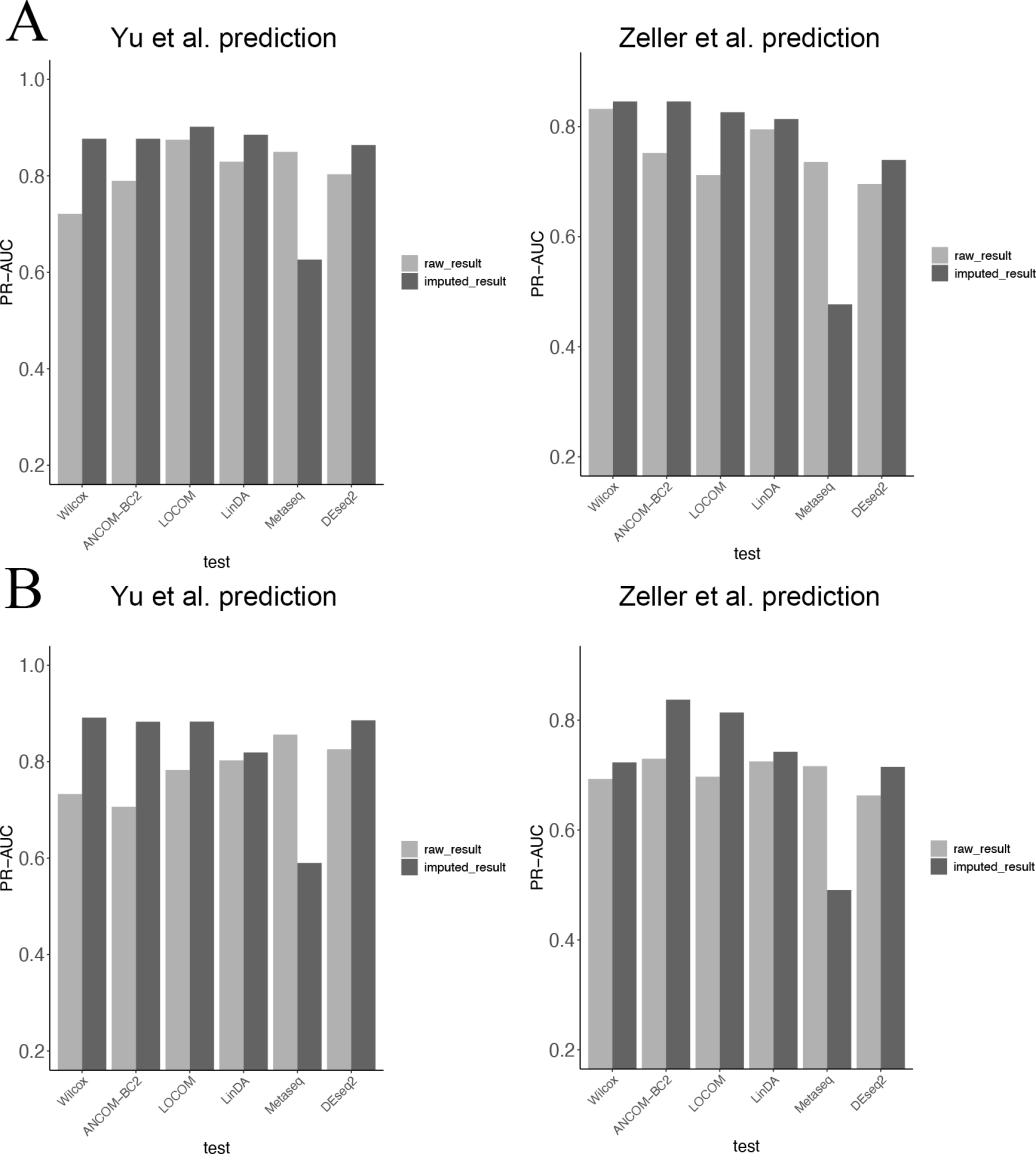
Accuracy of cross-prediction classification (PR-AUC) results using two classification algorithms for the two CRC datasets by Yu et al. [ **41] and Zeller et al. [42].** The left graph shows the predictive classification results for the Yu et al. [41] dataset using differentially abundant (DA) taxa as features obtained from Zeller et al. [42]‘s original dataset (light-colored bars) and the dataset imputed by TphPMF (dark-colored bars); the right graph displays the predictive classification results for the Zeller et al. [42] dataset using DA taxa as features obtained from Yu et al. [41]’s original dataset (light-colored bars) and the dataset imputed by TphPMF (dark-colored bars). **A.** Random Forest. **B.** XGBoost.

For the CRC datasets collected by Yu et al. [41] and Zeller et al. [42], we similarly used the DA taxa identified from the original dataset of Zeller et al. [42] and the TphPMF-imputed dataset using the aforementioned differential abundance analysis methods. We then proceeded to predict disease status in the Yu et al. [41] dataset using classifiers such as Random Forest and XGBoost. Continuing in a similar fashion, we compared the disease status prediction results for the Zeller et al. [42] dataset using DA taxa as features captured from both the original and imputed datasets of Yu et al. [41], again employing PR-AUC as the assessment metric. Fig 7A and 7B also demonstrate that TphPMF overall improves the accuracy of cross-prediction classification in most cases. These findings seem to imply that TphPMF holds potential for leveraging differential abundance analysis results from one dataset to predict the disease status in samples of another dataset within the same disease context.

## Discussion

In this article, we introduce a machine learning-based microbial data imputation method named TphPMF, which is based on Probabilistic Matrix Factorization and utilizes the phylogenetic relationships between microorganisms to construct Bayesian prior distributions. For missing data caused by various reasons, we employed a gamma-normal mixture modeling approach to accurately identify true non-biological zeros, thereby avoiding unnecessary errors in subsequent analyses due to the model’s handling of biological zeros. The incorporation of phylogenetic hierarchy information of taxa enables the model to utilize more meaningful prior information for posterior prediction of missing abundance values, significantly enhancing the imputation performance of the model. Additionally, the flexibility of our method is further enhanced by allowing researchers to adjust the model’s hyperparameters according to the specific situation, thereby broadening its applicability. Simulation studies set up based on real data confirmed that our method outperforms other existing imputation methods in terms of both performance and robustness.

In this study, we evaluated the performance of TphPMF in comparison to two other methods specifically designed for microbiome data analysis, mbImpute and mbDenoise. mbImpute constructs a linear regression model to impute missing data by leveraging information from similar taxa, similar microbiome samples, and sample covariates (when available). However, the relationships between similar taxa or samples are not necessarily linear, and in some cases, linear models may not be able to effectively impute missing data. mbDenoise utilizes information sharing between samples and taxonomic groups. By employing latent variable models, it denoises microbiome data so as to decouple the biological signal from technical variation. However, its model assumes that the samples are independent and identically distributed and fails to take into account the relationships among taxa, e.g., phylogenetic relationships. These assumptions are unreasonable, as the samples may not necessarily be independent. Meanwhile, the phylogenetic information of microorganisms can also offer valuable assistance for missing data imputation. In this approach, we employ a probability matrix factorization model and utilize both phylogenetic signals and structural matrix information as prior knowledge to impute missing data. We have taken into account the aspects that were neglected by the previous two methods, which is precisely the reason why our method can yield good results.

Differential abundance analysis of microbiomes is a critical tool in microbial ecology research. Through this analysis, we can gain insights into microbiological mechanisms underlying various human diseases, thereby informing new diagnostic and therapeutic strategies. Our research illustrates that integrating TphPMF with differential abundance analysis methods not only improves the accuracy of identifying differentially abundant (DA) taxa but also ensures that these taxa are relevant for disease prediction. Moreover, the application of TphPMF in conjunction with differential abundance analysis on real datasets has shown that it preserves the feature distribution of taxa non-zero abundance while enhancing disease prediction accuracy. Notably, our findings indicate that TphPMF consistently improves the accuracy of cross-prediction classification, suggesting its potential for significant research implications in future disease predictions across different datasets of the same disease type.

We have reason to believe that TphPMF can be used for imputation of most highly sparse microbiome taxon count matrices, effectively filling in missing data and removing certain obstacles for subsequent microbiome data analysis. However, the application of this method to other types of genomic data and more complex metagenomic data requires further exploration and research.

## Materials and methods

### Datasets

In this study, we utilized six real datasets from the R package curatedMetagenomicData [56]. These datasets comprised two Type 2 Diabetes (T2D) datasets (Qin et al. [38] and Karlsson et al. [36]) and four Colorectal Cancer (CRC) datasets(Zeller et al. [42], Yu et al. [41], Feng et al. [39] and Vogtmann et al. [40]). For each dataset, we downloaded the Operational Taxonomic Unit (OTU) data matrices, sample covariate data, and phylogenetic distance data of taxa.

Additionally, the robustness analysis utilized 16S rRNA sequencing data from 54 fecal samples of healthy individuals, provided by the R package HMP16SData [37].

### Data pre-processing

Given the significant variation in total counts across taxonomic count matrices from different microbiome datasets, we first normalized the count matrix using the following formula:


$${\text{O}}_{\text{ij}}^{\left(N\right)}=10^{6}\cdot \frac{{\text{O}}_{\text{ij}}}{\sum_{j^{\prime }=1}^{\text{M}}{\text{O}}_{\text{i}j^{\prime }}}$$

where ${\text{Ο}}^{\left(N\right)}=\left({\text{O}}_{\text{ij}}^{\left(N\right)}\right)\in {\text{R}}_{\geq 0}^{\text{N}\times \text{M}}$ represents the normalized taxonomic count matrix, with each sample’s total count scaling to $10^{6}$. Here, ${\text{O}}_{\text{ij}}$ represents the value of the $\left(\text{i},\text{j}\right)$ entry in the original microbial taxon count matrix, which contains M types of microbial taxa.

To meet the requirements of the TphPMF model, which necessitates a normally distributed dataset, we applied a logarithmic transformation to the normalized counts:


$${\text{Y}}_{\text{ij}}={\text{log}}_{10}\left({\text{O}}_{\text{ij}}^{\left(N\right)}+1.01\right)$$

where $\text{Y}=\left({\text{Y}}_{\text{ij}}\right)\in {\text{R}}_{>0}^{\text{N}\times \text{M}}$ denotes the matrix after normalization and log transformation. The addition of 1.01 ensures that ${\text{Y}}_{\text{ij}}>0$, facilitating subsequent analyses.

Furthermore, since TphPMF requires the input matrix to specify missing values as NA, we assigned NA to non-biological zero points in the microbiome taxonomic count matrix (i.e., the matrix values corresponding to ${\text{log}}_{10}\left(1.01\right)$ that require estimation). This adjustment ensures that TphPMF functions correctly.

### Identifying taxa abundance for estimation

Similar to the approach used in mbImpute [32] for identifying taxonomic groups whose abundance needs estimation, we initially employed an approximate binomial distribution one-tailed test. This test was used to pinpoint low-abundance but statistically significant taxa in the samples. Specifically, if the lower bound of the 95% confidence interval for the proportion of non-low-abundance taxa in the samples exceeds zero, the taxa are considered statistically significant; otherwise, they are excluded.

To identify the taxa abundance that represents non-biological zeros, we applied a mixture model to simulate the abundance distribution of each taxa in the count matrix [32]:


$${\text{Y}}_{\text{ij}}\sim {\text{p}}_{\text{j}}\cdot \text{Γ}\left({\text{α}}_{\text{j}},{\text{β}}_{\text{j}}\right)+\left(1-{\text{p}}_{\text{j}}\right)\cdot N\left({\text{X}}_{\text{i}\cdot }^{\text{T}}{\text{γ}}_{\text{j}},{\text{σ}}_{\text{j}}^{2}\right)$$

here, the abundance ${\text{Y}}_{\text{ij}}$ of a microbe follows a gamma distribution $\text{Γ}\left({\text{α}}_{\text{j}},{\text{β}}_{\text{j}}\right)$ with probability ${\text{p}}_{\text{j}}$, and a normal distribution with mean ${\text{X}}_{\text{i}\cdot }^{\text{T}}{\text{γ}}_{\text{j}}$ and standard deviation ${\text{σ}}_{\text{j}}$ with probability $\left(1-{\text{p}}_{\text{j}}\right)$. The vector ${\text{X}}_{\text{i}\cdot }^{\text{T}}\in {\text{R}}^{\text{q}}$ represents the covariates of the i-th sample, ${\text{γ}}_{\text{j}}\in {\text{R}}^{\text{q}}$ represents the effects of covariates on the abundance of taxa j, and the standard deviation ${\text{σ}}_{\text{j}}>0$. The parameters of the model are estimated using the EM algorithm. For those taxa with a p-value ≤0.05 in the likelihood ratio test (LRT), whether ${\text{Y}}_{\text{ij}}$ requires estimation is determined by the estimated posterior probability that ${\text{Y}}_{\text{ij}}$ originates from the gamma distribution component of the mixture model:


$${\text{e}}_{\text{ij}}=\frac{\hat{{\text{p}}_{\text{j}}}\cdot {\text{f}}_{\text{Γ}}\left({\text{Y}}_{\text{ij}};\hat{{\text{α}}_{\text{j}}},\hat{{\text{β}}_{\text{j}}}\right)}{\hat{{\text{p}}_{\text{j}}}\cdot {\text{f}}_{\text{Γ}}\left({\text{Y}}_{\text{ij}};\hat{{\text{α}}_{\text{j}}},\hat{{\text{β}}_{\text{j}}}\right)+\left(1-\hat{{\text{p}}_{\text{j}}}\right)\cdot {\text{f}}_{N}\left({\text{Y}}_{\text{ij}};{\text{X}}_{\text{i}\cdot }^{\text{T}}\hat{{\text{γ}}_{\text{j}}},{\hat{{\text{σ}}_{\text{j}}}}^{2}\right)}$$

where ${\text{f}}_{\text{Γ}}$ and ${\text{f}}_{N}$ are the probability density functions of the gamma and normal components of the mixture model, respectively. If ${\text{e}}_{\text{ij}}\geq 0.5$, we designate the corresponding ${\text{Y}}_{\text{ij}}$ as a non-biological zero.

### Generating phylogenetic hierarchy information matrix

The phylogenetic distance between microbes illustrates their evolutionary relationships, with shorter distances suggesting a closer kinship and greater similarity. We utilized a complete linkage clustering method to hierarchically organize the microbes based on their phylogenetic distances, categorizing them into three levels: “ S “, “ G “, and “ F “. This process resulted in a phylogenetic hierarchy information matrix. The thresholds for cutting the clustering tree at these three levels were treated as hyperparameters, adjustable based on the variability in phylogenetic distances observed in different datasets.

### Estimating missing taxa abundance

**Fig 8:**
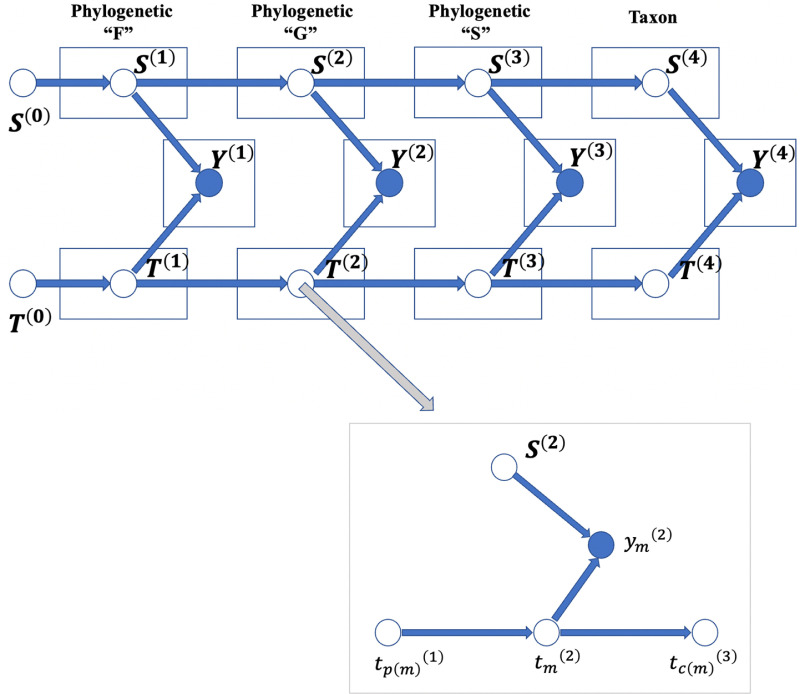
Schematic of the TphPMF model. Here, Y represents the microbial taxa count matrix, S denotes the latent vector matrix corresponding to the sample side of the count matrix, and T represents the latent vector matrix corresponding to the entities (taxa) side. Numbers in parentheses indicate different phylogenetic hierarchy levels. The gray inset in the lower right corner shows the operation mode of the Gibbs sampler, where p(m) is the parent node of m in the previous layer, and c(m) is the collection of child nodes of m in the lower layer.

**Fig 9:**
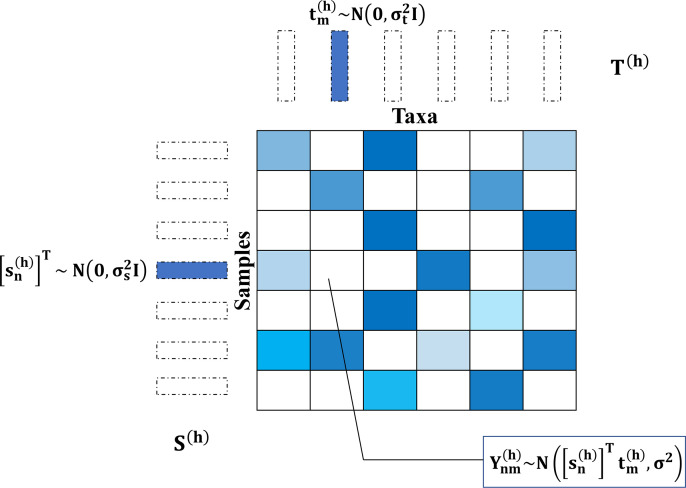
The schematic diagram of the principle of TphPMF imputing non-biological zeros at each hierarchical level. Here, the blank areas represent non-biological zeros that need to be imputed in the taxon count matrix. ${\text{t}}_{\text{m}}^{\left(\text{h}\right)}$ denotes the latent vector on the taxon side at the hierarchical level h, and ${\text{s}}_{\text{n}}^{\left(\text{h}\right)}$ denotes the latent vector on the sample side, both of which have a Gaussian normal distribution with a mean of 0. Each missing taxon abundance ${\text{Y}}_{\text{nm}}^{\left(\text{h}\right)}$ can be approximated by the product of ${\left[{\text{s}}_{\text{n}}^{\left(\text{h}\right)}\right]}^{\text{T}}$ and ${\text{t}}_{\text{m}}^{\left(\text{h}\right)}$.

In the taxa count matrix $\text{Y}\in {\text{R}}_{>0}^{\text{N}\times \text{M}}$, each row and column represent a sample (S) and a microbe taxon (T), respectively. Based on the traditional PMF method [34], TphPMF uses the latent vector information from the adjacent hierarchical level (h) as prior information for the current level to perform posterior estimation of the missing taxa abundances (Fig 8). The prior distributions of the latent vectors (s and t) for the count matrix are defined as Gaussian normal distributions: $N\left(0,{\text{σ}}_{\text{s}}^{2}\text{Ι}\right)$, $N\left(0,{\text{σ}}_{\text{t}}^{2}\text{Ι}\right)$, with ${\text{Y}}_{\text{ij}}\sim N\left({\text{s}}_{\text{i}}^{\text{T}}{\text{t}}_{\text{j}},{\text{σ}}^{2}\right)$. The matrices formed by stacking the latent vectors s and t at the hierarchy level h are denoted as ${\text{S}}^{\left(\text{h}\right)}$ and ${\text{T}}^{\left(\text{h}\right)}$, respectively. We ran the Gibbs sampler [57] iteratively in a top-down and bottom-up manner to optimize and update ${\text{S}}^{\left(\text{h}\right)}$ and ${\text{T}}^{\left(\text{h}\right)}$ (refer to S1 Table). At each hierarchical level, the principle of TphPMF imputing non-biological zeros is illustrated in Fig 9. To estimate the model parameters, the posterior distribution of ${\text{S}}^{\left(\text{h}\right)}$ and ${\text{T}}^{\left(\text{h}\right)}$ is defined as a posterior probability model:

$\text{p}\left(\left\{{\text{S}}^{\left(\text{h}\right)}\right\},\left\{{\text{T}}^{\left(\text{h}\right)}\right\}\text{|}\left\{{\text{Y}}^{\left(\text{h}\right)}\right\},{\text{σ}}^{2},{\text{S}}^{\left(0\right)},{\text{T}}^{\left(0\right)}\right)\propto \overset{\text{H}}{\underset{\text{h}=1}{\prod }}\left\{\underset{\text{n}}{\prod }N\left({\text{s}}_{\text{n}}^{\left(\text{h}\right)}{\text{|s}}_{\text{n}}^{\left(\text{h}-1\right)},{\text{σ}}_{\text{s}}^{2}\text{Ι}\right)\underset{\text{m}}{\prod }N\left({\text{t}}_{\text{m}}^{\left(\text{h}\right)}{\text{|t}}_{\text{p}\left(\text{m}\right)}^{\left(\text{h}-1\right)},{\text{σ}}_{\text{t}}^{2}\text{Ι}\right)\underset{\text{n},\text{m}}{\prod }{\text{δ}}_{\text{nm}}^{\left(\text{h}\right)}N\left({\text{y}}_{\text{nm}}^{\left(\text{h}\right)}{\text{|s}}_{\text{n}}^{\left(\text{h}\right)},{\text{t}}_{\text{m}}^{\left(\text{h}\right)},{\text{σ}}^{2}\right)\right\}$ here, ${\text{δ}}_{\text{nm}}^{\left(\text{h}\right)}=1$ when the entry $\left(\text{n},\text{m}\right)$ of ${\text{Y}}^{\left(\text{h}\right)}$ is not missing, otherwise it is 0; $\text{p}\left(\text{m}\right)$ is the parent node of m at the upper layer, for example, if m is a microbe, then $\text{p}\left(\text{m}\right)$ represents the taxa that m belongs to at the “S” level. We aimed to maximize the posterior log of the above probability model to achieve maximum a posteriori inference, which ultimately boils down to minimizing the regularized squared loss. At each level, the objective function becomes:


$${\text{E}}^{\left(\text{h}\right)}=\underset{\text{n},\text{m}}{\sum }{\text{δ}}_{\text{nm}}^{\left(\text{h}\right)}{\text{y}}_{\text{nm}}^{\left(\text{h}\right)}-{\text{s}}_{\text{n}}^{\left(\text{h}\right)},{\text{t}}_{\text{m}}^{\left(\text{h}\right)}{}_{2}^{2}+{\text{λ}}_{\text{t}}\underset{\text{m}}{\sum }\left({\text{t}}_{\text{m}}^{\left(\text{h}\right)}-{\text{t}}_{\text{p}\left(\text{m}\right)}^{\left(\text{h}-1\right)}{}_{2}^{2}+{\text{Ι}}_{\left(\text{h}<\text{H}\right)}\underset{m^{\prime }\in \text{c}\left(\text{m}\right)}{\sum }{\text{t}}_{\text{m}}^{\left(\text{h}\right)}-{\text{t}}_{m^{\prime }}^{\left(\text{h}+1\right)}{}_{2}^{2}\right)+{\text{λ}}_{\text{s}}\underset{\text{n}}{\sum }\left({\text{s}}_{\text{n}}^{\left(\text{h}\right)}-{\text{s}}_{\text{n}}^{\left(\text{h}-1\right)}{}_{2}^{2}+{\text{Ι}}_{\left(\text{h}<\text{H}\right)}{\text{s}}_{\text{n}}^{\left(\text{h}\right)}-{\text{s}}_{\text{n}}^{\left(\text{h}+1\right)}{}_{2}^{2}\right)$$

where ${\text{λ}}_{\text{t}}={\text{σ}}^{2}/{\text{σ}}_{\text{t}}^{2}$ and ${\text{λ}}_{\text{s}}={\text{σ}}^{2}/{\text{σ}}_{\text{s}}^{2}$; $\text{c}\left(\text{m}\right)$ is the set of child nodes of m at the lower level, for instance, when h=1, $\text{c}\left(\text{m}\right)$ represents the collection of all microbes that belong to the same taxa as microbe m at the “ S “ level; ${\text{Ι}}_{\left(\text{h}<\text{H}\right)}$ is an indicator function that takes the value 1 when h<H, and 0 otherwise.

### Hyperparameter tuning

Setting appropriate hyperparameters is crucial for optimizing the performance of the TphPMF model for microbiome data imputation. Adjustments to the model’s hyperparameters, including the number of cross-validations (defaulted to 10), the number of hierarchical levels used for imputing missing values (defaulted to the total number of levels), the total number of samples produced by the Gibbs sampler at each fold level, the number of initial sampling parameters discarded, the gap between retained sampling parameters, the size of the latent vectors, and the number of cross-validation folds to adjust (defaulted to 10) as well as different heights across three hierarchical levels to cut the clustering tree when generating the phylogenetic hierarchical information matrix for microorganisms, are necessary based on specific dataset characteristics, task requirements, and empirical performance measures such as RMSE. We compared the MSE between the imputed datasets and the complete datasets across four key parameter variations to guide our selection of parameter values. The results are presented in S2 Table.

## Supporting information

S1 Fig Comparison of the performance of TphPMF with other imputation methods in Simulation 3.
(TIF)

S2 Fig Comparison of the performance of TphPMF with other imputation methods in three Simulations (zero proportions: Simulation1: 62.8%; Simulation2: 68.5%; Simulation3: 69.6%).
A-B. The mean squared error (MSE) and the mean Pearson correlation between imputed and complete data across all taxa in Simulation 1. **C-D.** Results of Simulation 2. **E-F.** Results of Simulation 3.
(TIF)

S3 Fig Comparison of the performance of TphPMF with other imputation methods in three Simulations (zero proportions: Simulation1: 71.5%; Simulation2: 74.3%; Simulation3: 75.1%).
A-B. The mean squared error (MSE) and the mean Pearson correlation between imputed and complete data across all taxa in Simulation 1. **C-D.** Results of Simulation 2. **E-F.** Results of Simulation 3.
(TIF)

S4 Fig Comparison of the performance of TphPMF with other imputation methods in three Simulations (zero proportions: Simulation1: 75.5%; Simulation2: 76.9%; Simulation3: 77.9%).
A-B. The mean squared error (MSE) and the mean Pearson correlation between imputed and complete data across all taxa in Simulation 1. **C-D.** Results of Simulation 2. **E-F.** Results of Simulation 3.
(TIF)

S5 Fig Comparison of runtime between TphPMF, mbImpute, and mbDenoise.
(TIF)

S6 Fig Precision, recall, and F1 score of six DA methods with and without imputation using mbImpute, mbDenoise or TphPMF in the case of 20 DA taxa.
A. Wilcoxon rank-sum test. **B.** ANCOM-BC2. **C.** metagenomeSeq. **D.** DESeq2-phyloseq. **E.** LOCOM. **F.** LinDA.
(TIF)

S7 Fig Precision, recall, and F1 score of six DA methods with and without imputation using mbImpute, mbDenoise or TphPMF in the case of 45 DA taxa.
A. Wilcoxon rank-sum test. **B.** ANCOM-BC2. **C.** metagenomeSeq. **D.** DESeq2-phyloseq. **E.** LOCOM. **F.** LinDA.
(TIF)

S8 Fig Abundance distribution of four example taxa after the introduction of two outlier samples.
A-D represent four different taxa, respectively. The top three graphs show the abundance distribution before imputation, while the bottom three graphs show the abundance distribution after imputation.
(TIF)

S9 Fig Distribution of taxon p-values calculated by ANCOM-BC2, DESeq2_phyloseq and LOCOM before and after imputation with TphPMF.
(A) ANCOM-BC2. (B) DESeq2_phyloseq. **(C)** LOCOM.
(TIF)

S10 Fig Results of sample disease state prediction using XGBoost algorithm with DA taxa identified by DESeq2-phyloseq as features, before and after imputation by TphPMF.
(TIF)

S11 Fig The abundance distribution of three example taxa identified by DESeq2-phyloseq before imputation with TphPMF and not identified after imputation in the dataset of Yu et al. [41] and the dataset of Zeller et al. [42]. A. In the dataset of Yu et al. [41], and the upper three graphs represent the distribution before imputation, the lower three graphs represent the distribution after imputation. **B.** In the dataset of Zeller et al. [42], and the upper three graphs represent the distribution before imputation, the lower three graphs represent the distribution after imputation.
(TIF)

S12 Fig Comparison of Spearman correlations between the full-sample abundances and the original non-zero sample abundances before and after imputation with TphPMF, using Spearman correlation to define similarity, for all samples, “disease” samples, and control samples in 2 T2D datasets and 4 CRC datasets.
Light-colored bars represent the Spearman correlation between the original full-sample and original non-zero sample abundances, while dark-colored bars represent the Spearman correlation between the imputed full-sample and original non-zero sample abundances.
(TIF)

S13 Fig Accuracy of cross-prediction classification (PR-AUC) results using two classification algorithms for the two T2D datasets by Qin et al. [38] and Karlsson et al. [36].
The left graph shows the predictive classification results for the Qin et al. [38] dataset using differentially abundant (DA) taxa as features obtained from Karlsson et al. [36]‘s original dataset (light-colored bars) and the dataset imputed by TphPMF (dark-colored bars); the right graph displays the predictive classification results for the Karlsson et al. [36] dataset using DA taxa as features obtained from Qin et al. [38]’s original dataset (light-colored bars) and the dataset imputed by TphPMF (dark-colored bars). A. Linear kernel Support Vector Machine (SVM). B. Gaussian kernel Support Vector Machine (SVM).
(TIF)

S1 Table Algorithm for iterative sampling in the Gibbs sampler.
Here, h represents the phylogenetic hierarchy of the taxa, s denotes the samples (rows of the matrix), t denotes the taxa (columns of the matrix), p(m) represents the parent node, and c(m) represents the child node.
(PDF)

S2 Table Comparison of the MSE between the TphPMF-imputed and complete datasets across four key parameter variations in Simulation 1.
(PDF)

S1 File The details of three simulations comparing TphPMF with several existing popular genomic data estimation methods for recovering missing taxonomic abundances.
(DOCX)
