# Supplementary figures and images for: TphPMF: A microbiome data imputation method using hierarchical Bayesian Probabilistic Matrix Factorization
Source: PLoS Comput Biol. 2025 Mar 11;21(3):e1012858. doi: 10.1371/journal.pcbi.1012858 (PMC11957397; doi:10.1371/journal.pcbi.1012858)

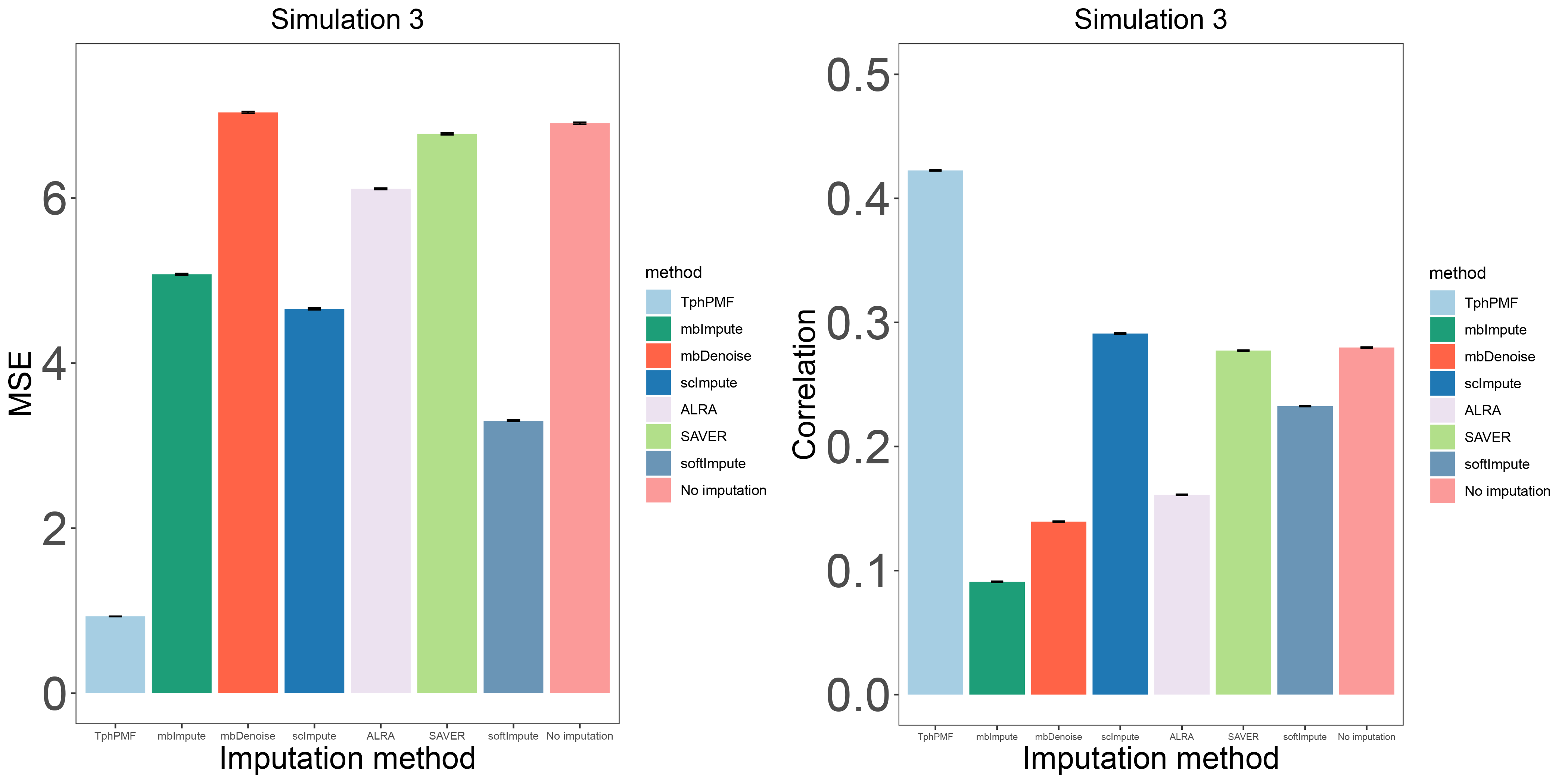

Supplement: S1 Fig — (TIF) [file pcbi.1012858.s001.tif]

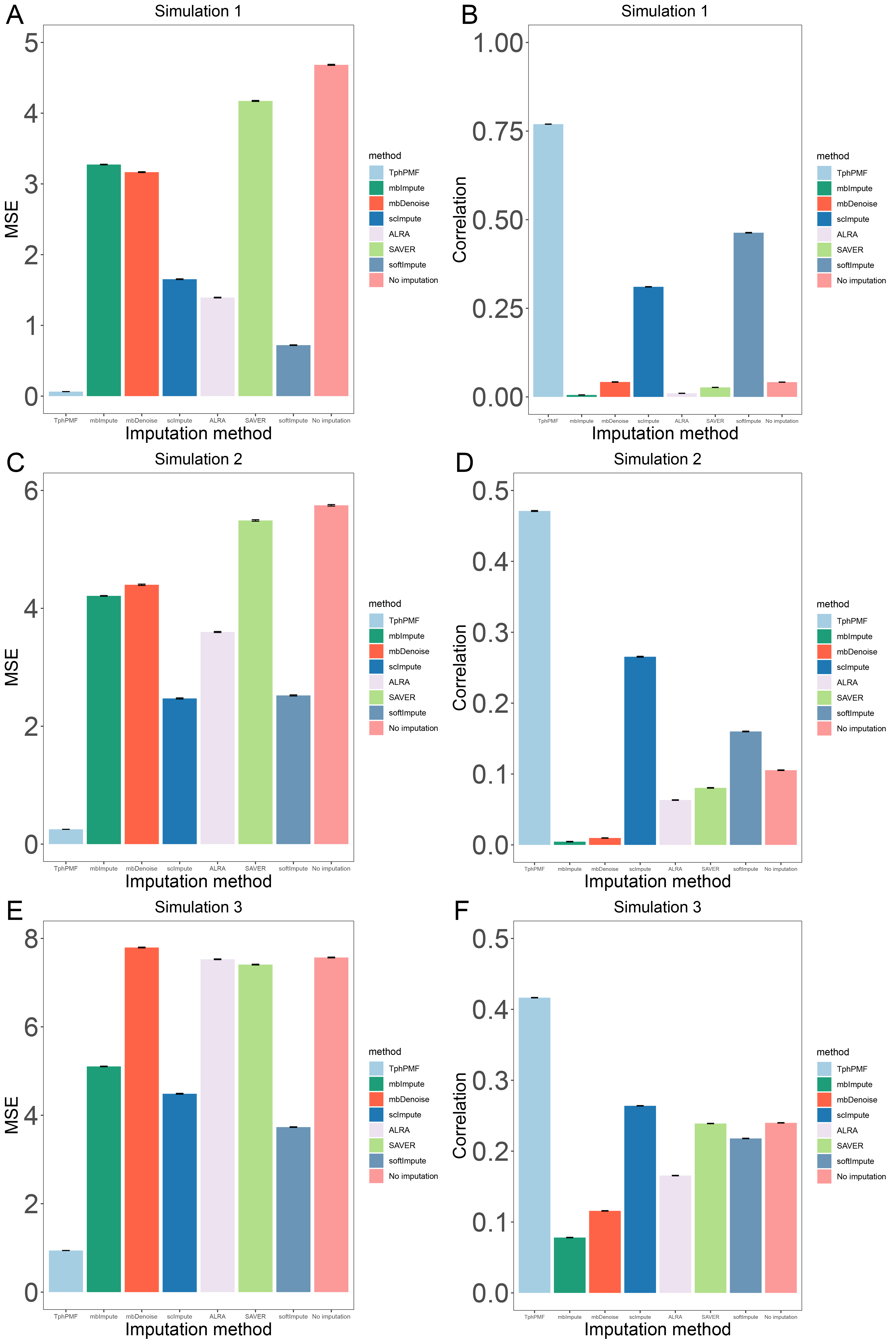

Supplement: S2 Fig — A-B. The mean squared error (MSE) and the mean Pearson correlation between imputed and complete data across all taxa in Simulation 1. C-D. Results of Simulation 2. E-F. Results of Simulation 3. (TIF) [file pcbi.1012858.s002.tif]

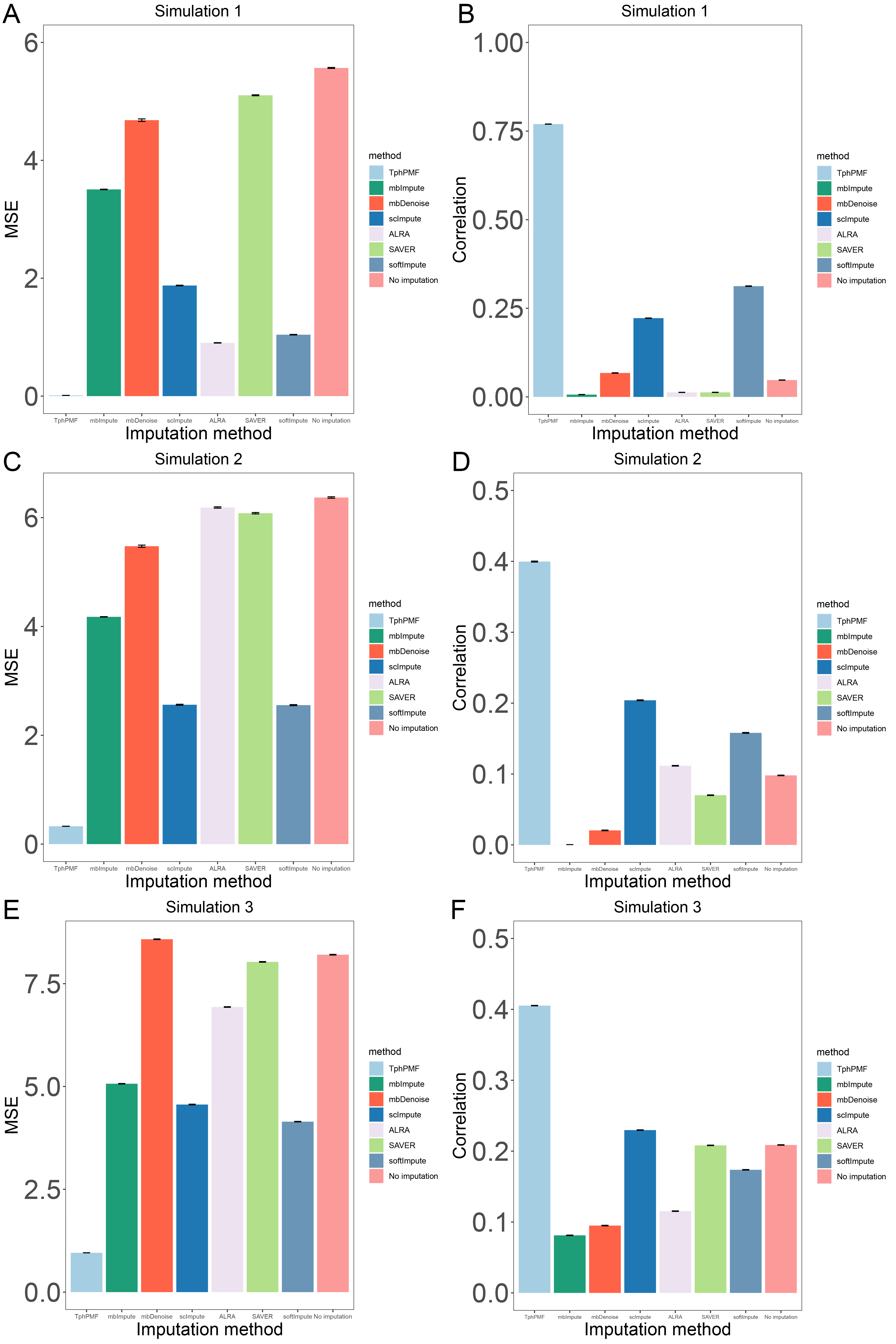

Supplement: S3 Fig — A-B. The mean squared error (MSE) and the mean Pearson correlation between imputed and complete data across all taxa in Simulation 1. C-D. Results of Simulation 2. E-F. Results of Simulation 3. (TIF) [file pcbi.1012858.s003.tif]

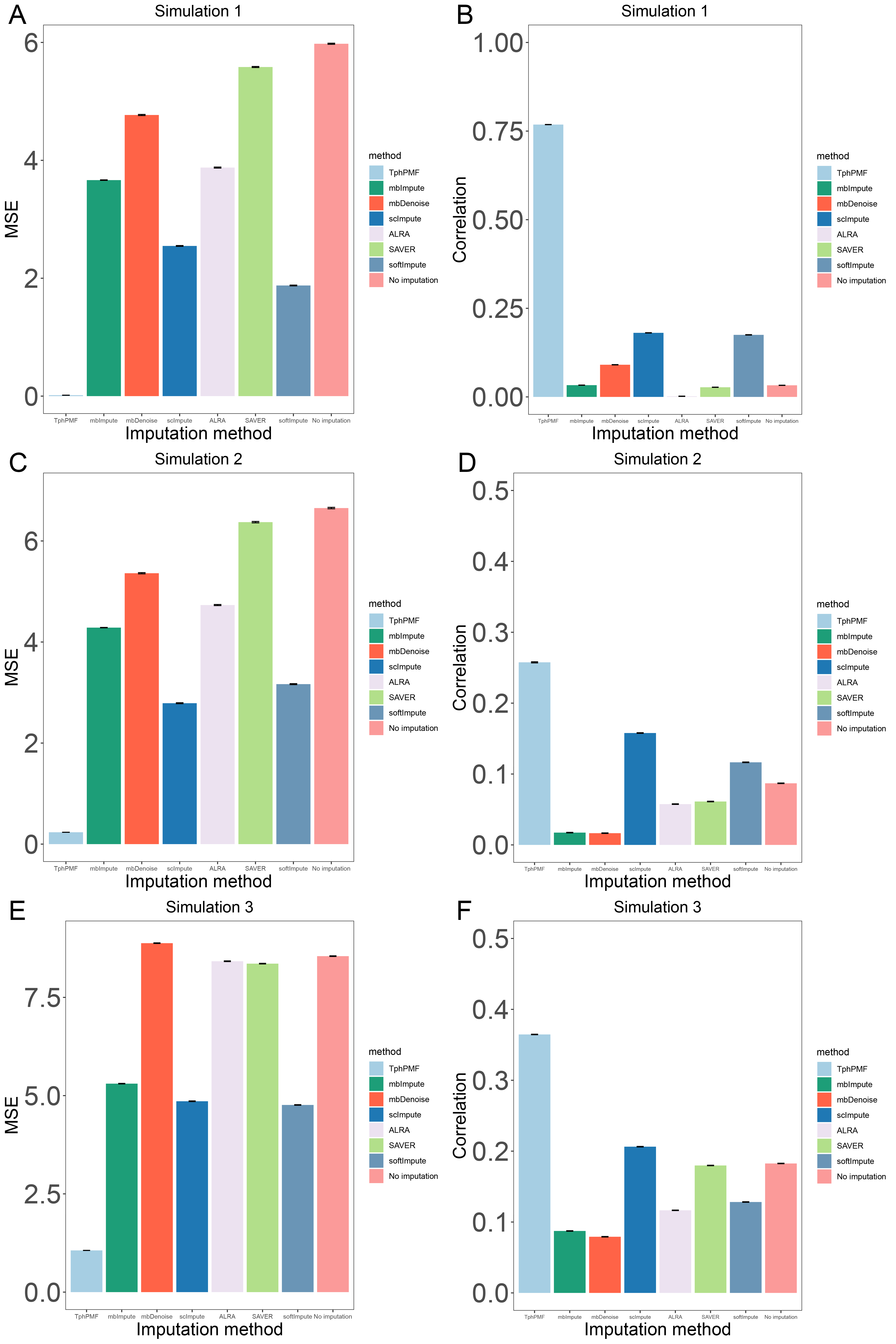

Supplement: S4 Fig — A-B. The mean squared error (MSE) and the mean Pearson correlation between imputed and complete data across all taxa in Simulation 1. C-D. Results of Simulation 2. E-F. Results of Simulation 3. (TIF) [file pcbi.1012858.s004.tif]

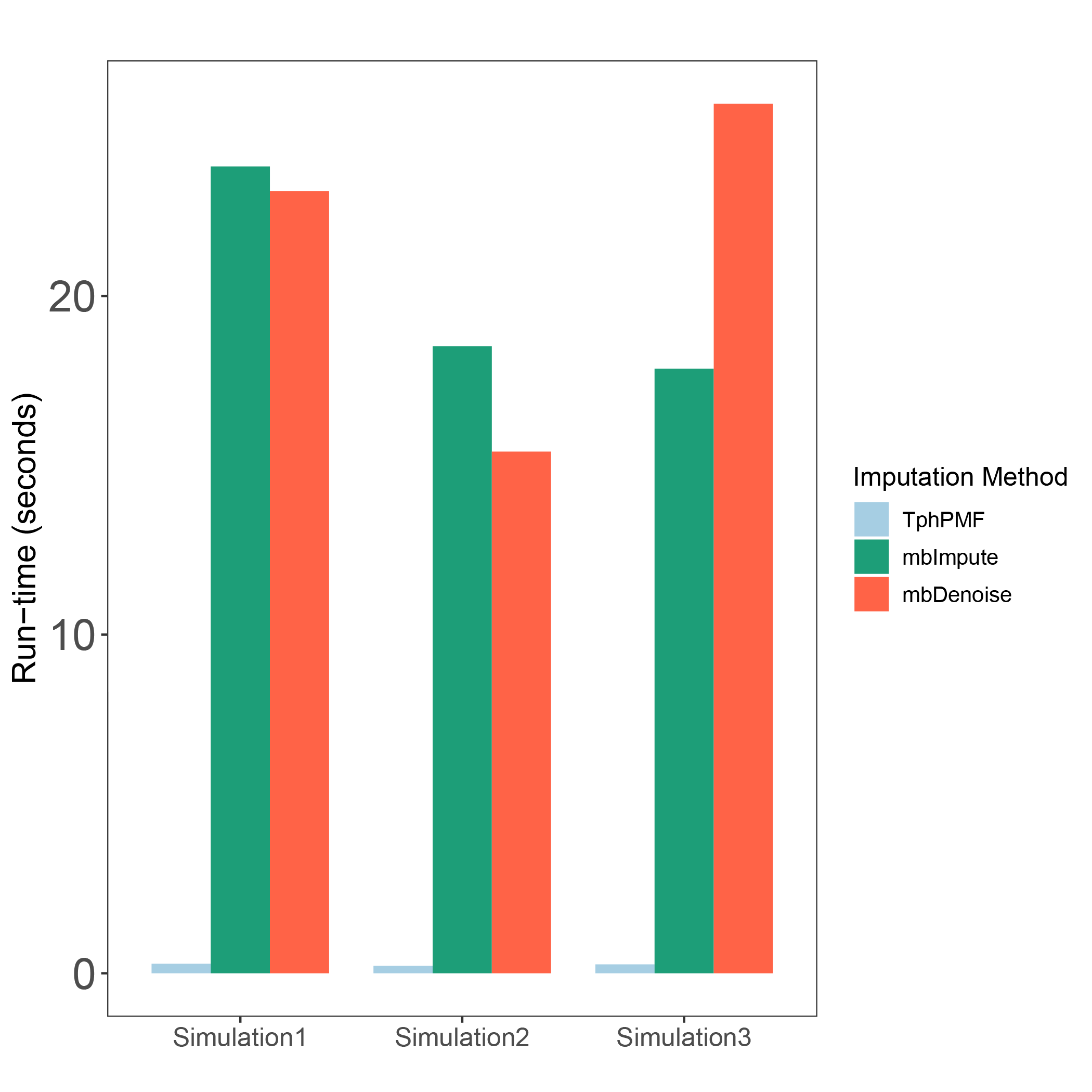

Supplement: S5 Fig — (TIF) [file pcbi.1012858.s005.tif]

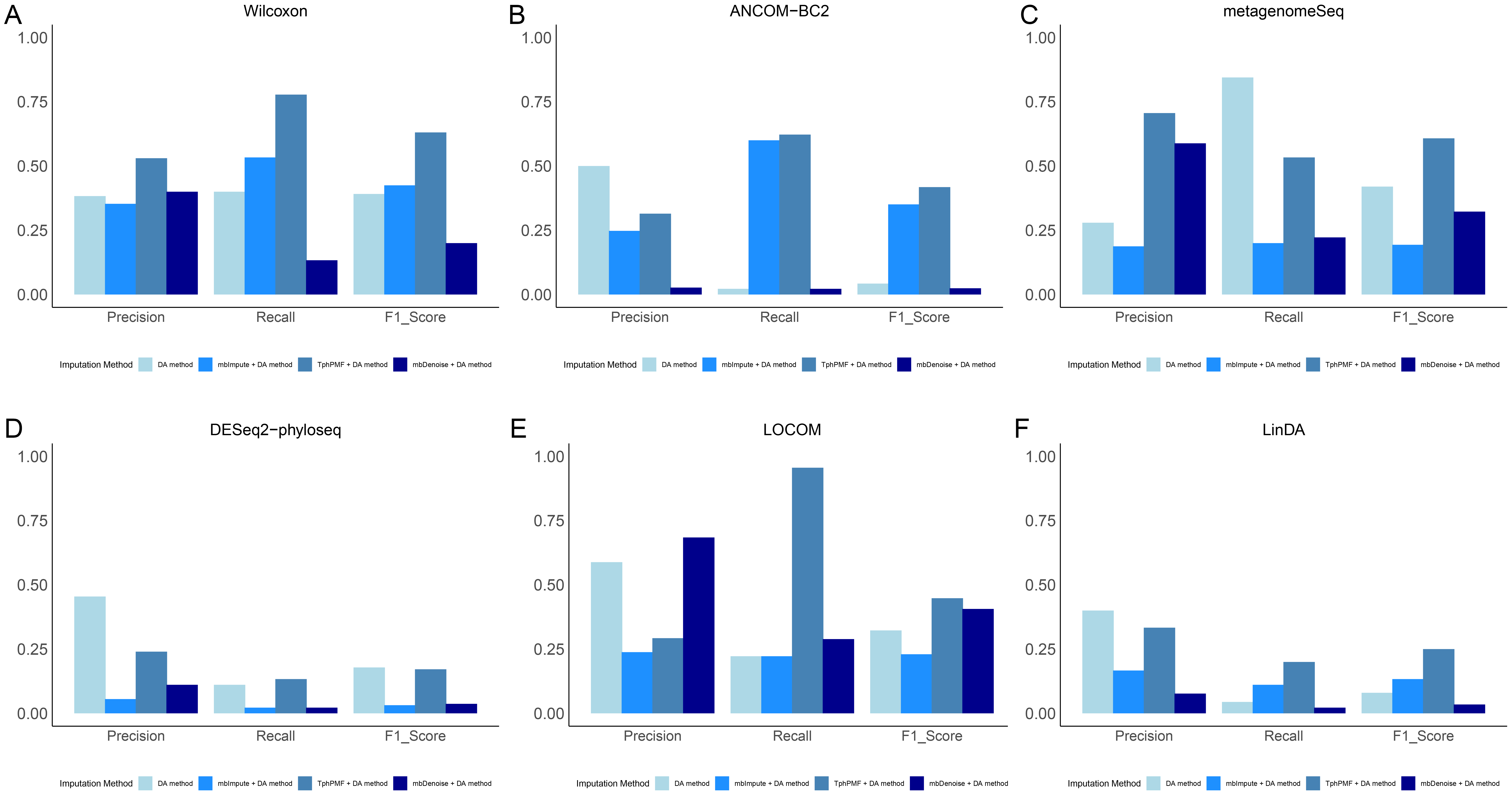

Supplement: S6 Fig — A. Wilcoxon rank-sum test. B. ANCOM-BC2. C. metagenomeSeq. D. DESeq2-phyloseq. E. LOCOM. F. LinDA. (TIF) [file pcbi.1012858.s006.tif]

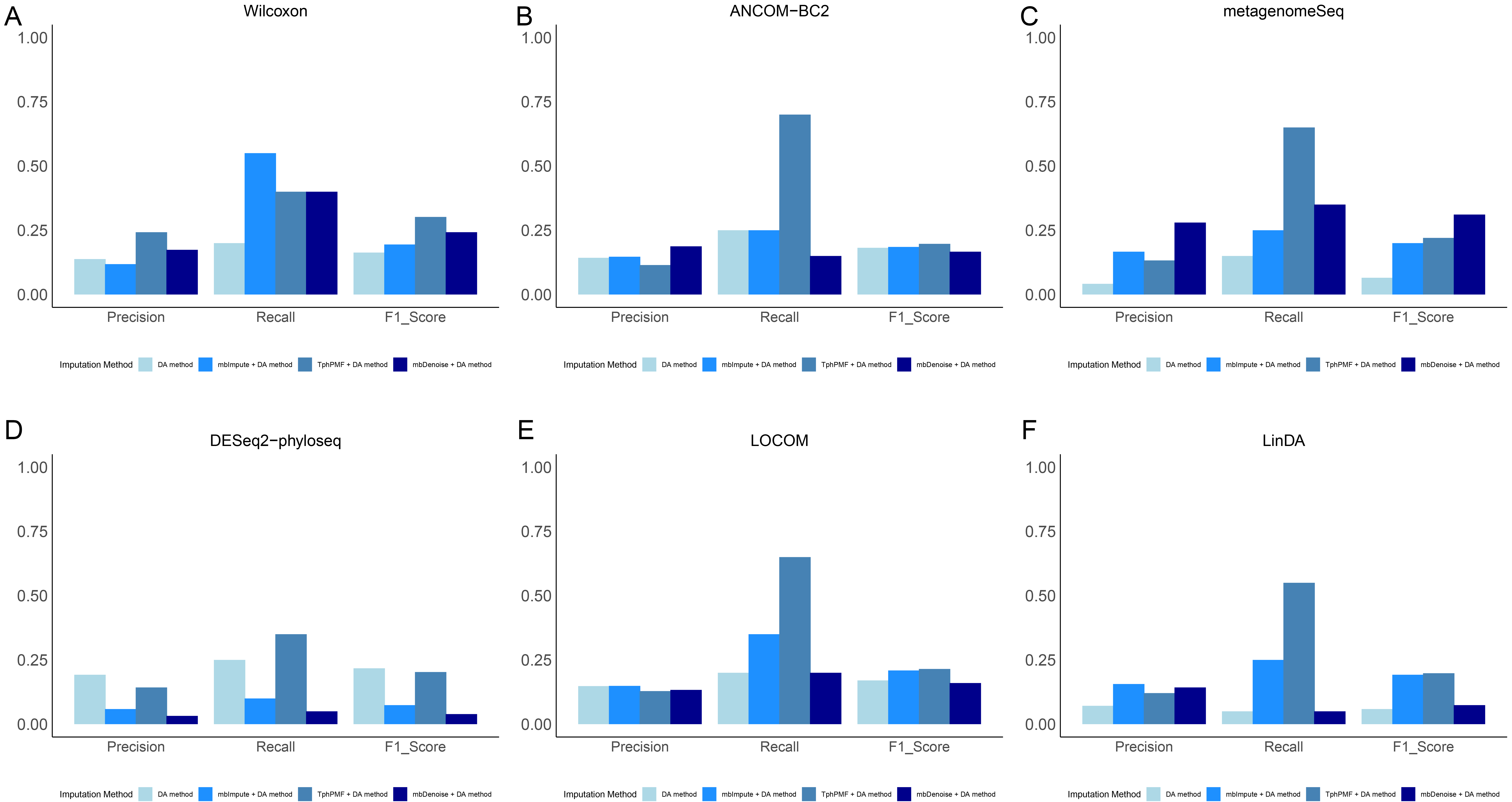

Supplement: S7 Fig — A. Wilcoxon rank-sum test. B. ANCOM-BC2. C. metagenomeSeq. D. DESeq2-phyloseq. E. LOCOM. F. LinDA. (TIF) [file pcbi.1012858.s007.tif]

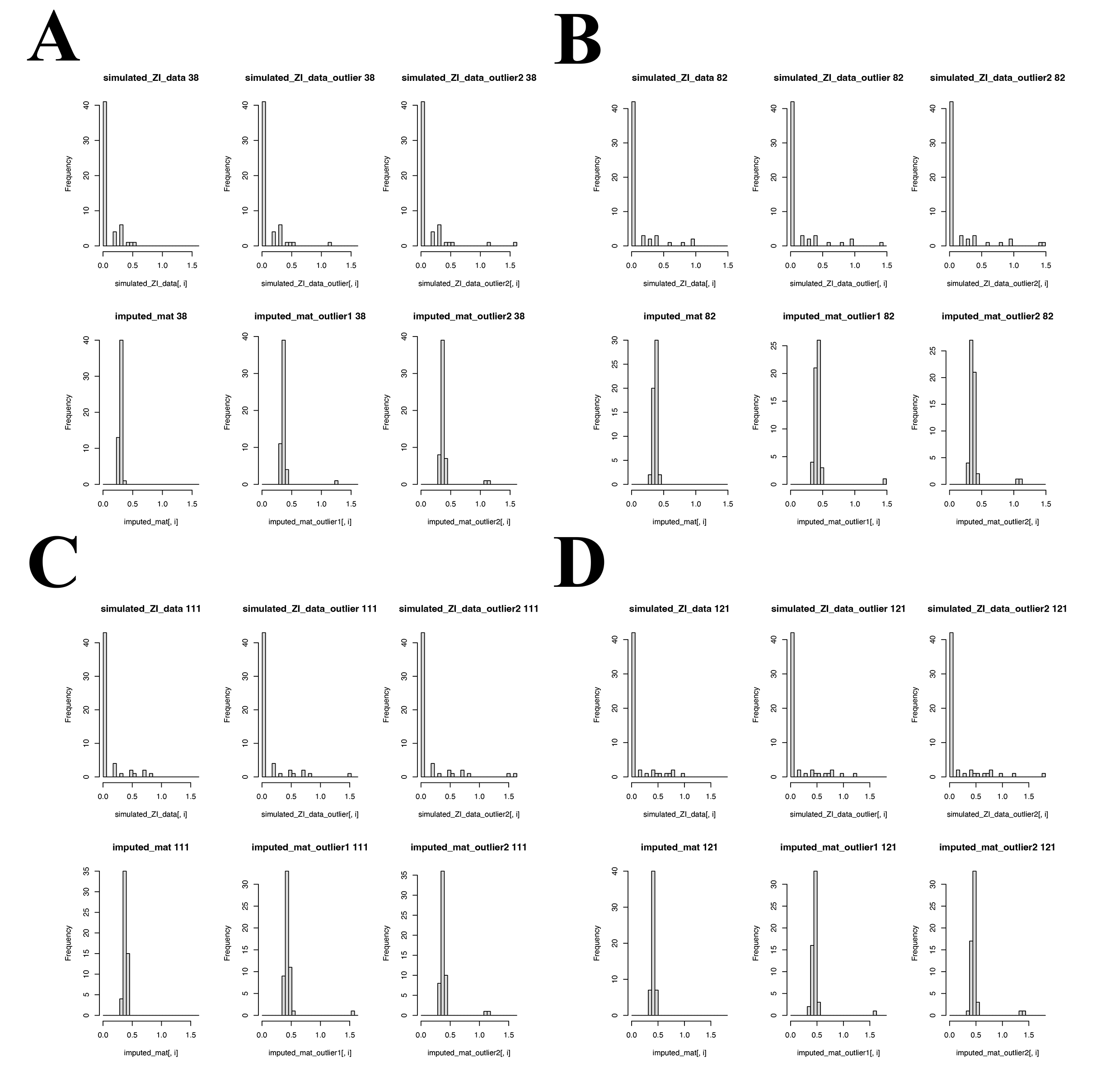

Supplement: S8 Fig — A-D represent four different taxa, respectively. The top three graphs show the abundance distribution before imputation, while the bottom three graphs show the abundance distribution after imputation. (TIF) [file pcbi.1012858.s008.tif]

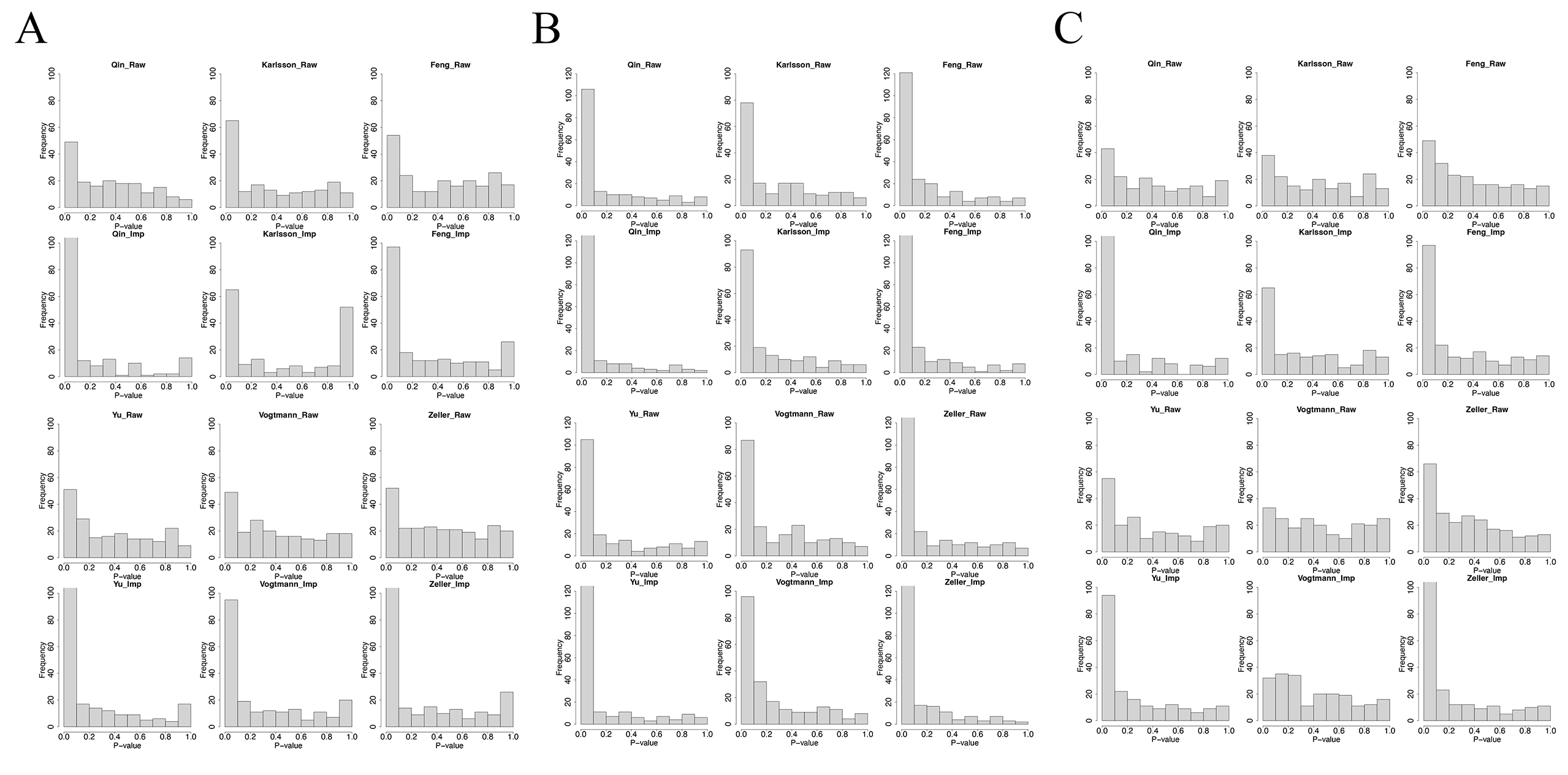

Supplement: S9 Fig — (A) ANCOM-BC2. (B) DESeq2_phyloseq. (C) LOCOM. (TIF) [file pcbi.1012858.s009.tif]

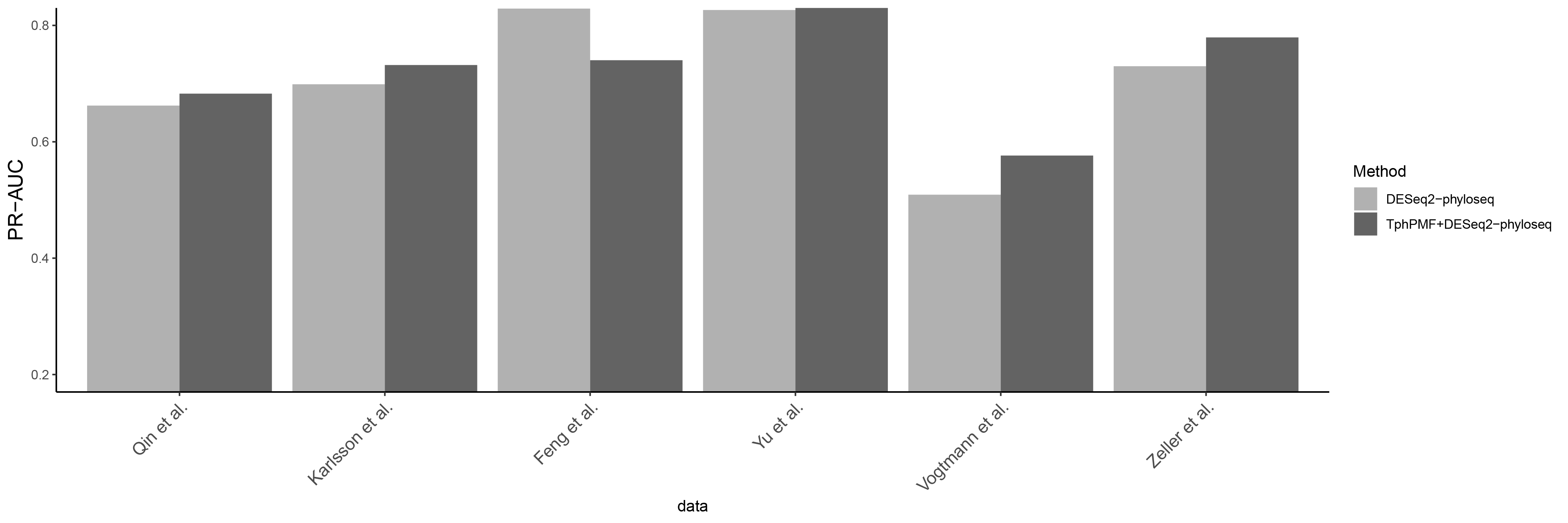

Supplement: S10 Fig — (TIF) [file pcbi.1012858.s010.tif]

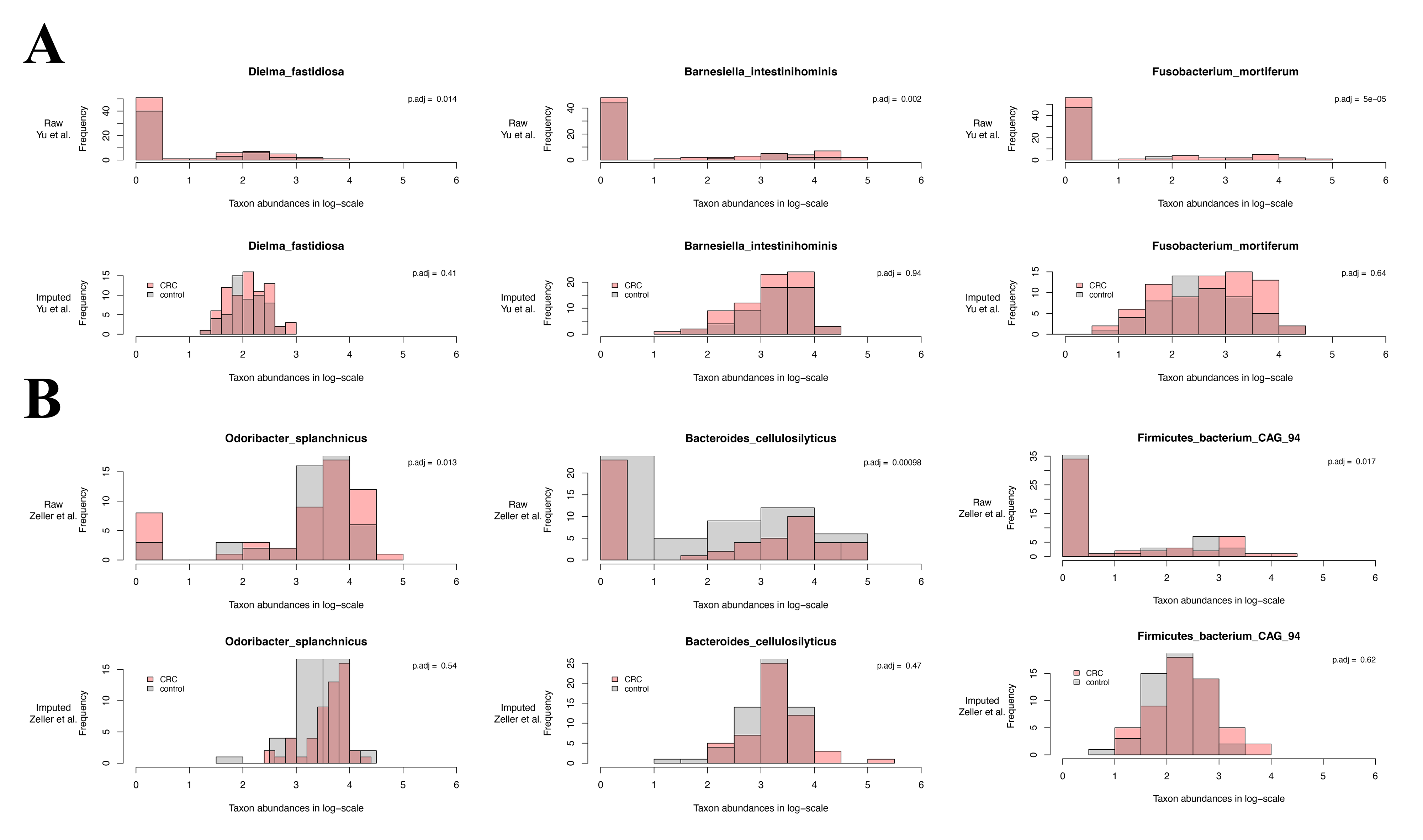

Supplement: S11 Fig — A. In the dataset of Yu et al. [41], and the upper three graphs represent the distribution before imputation, the lower three graphs represent the distribution after imputation. B. In the dataset of Zeller et al. [42], and the upper three graphs represent the distribution before imputation, the lower three graphs represent the distribution after imputation. (TIF) [file pcbi.1012858.s011.tif]

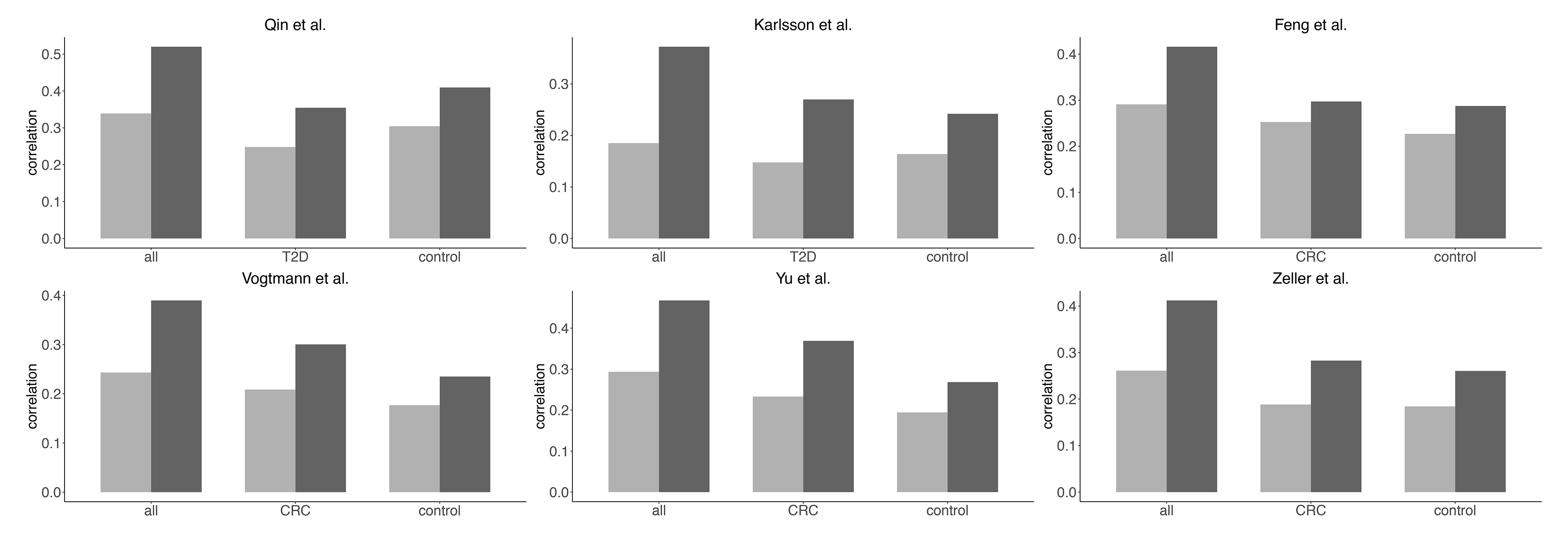

Supplement: S12 Fig — Light-colored bars represent the Spearman correlation between the original full-sample and original non-zero sample abundances, while dark-colored bars represent the Spearman correlation between the imputed full-sample and original non-zero sample abundances. (TIF) [file pcbi.1012858.s012.tif]

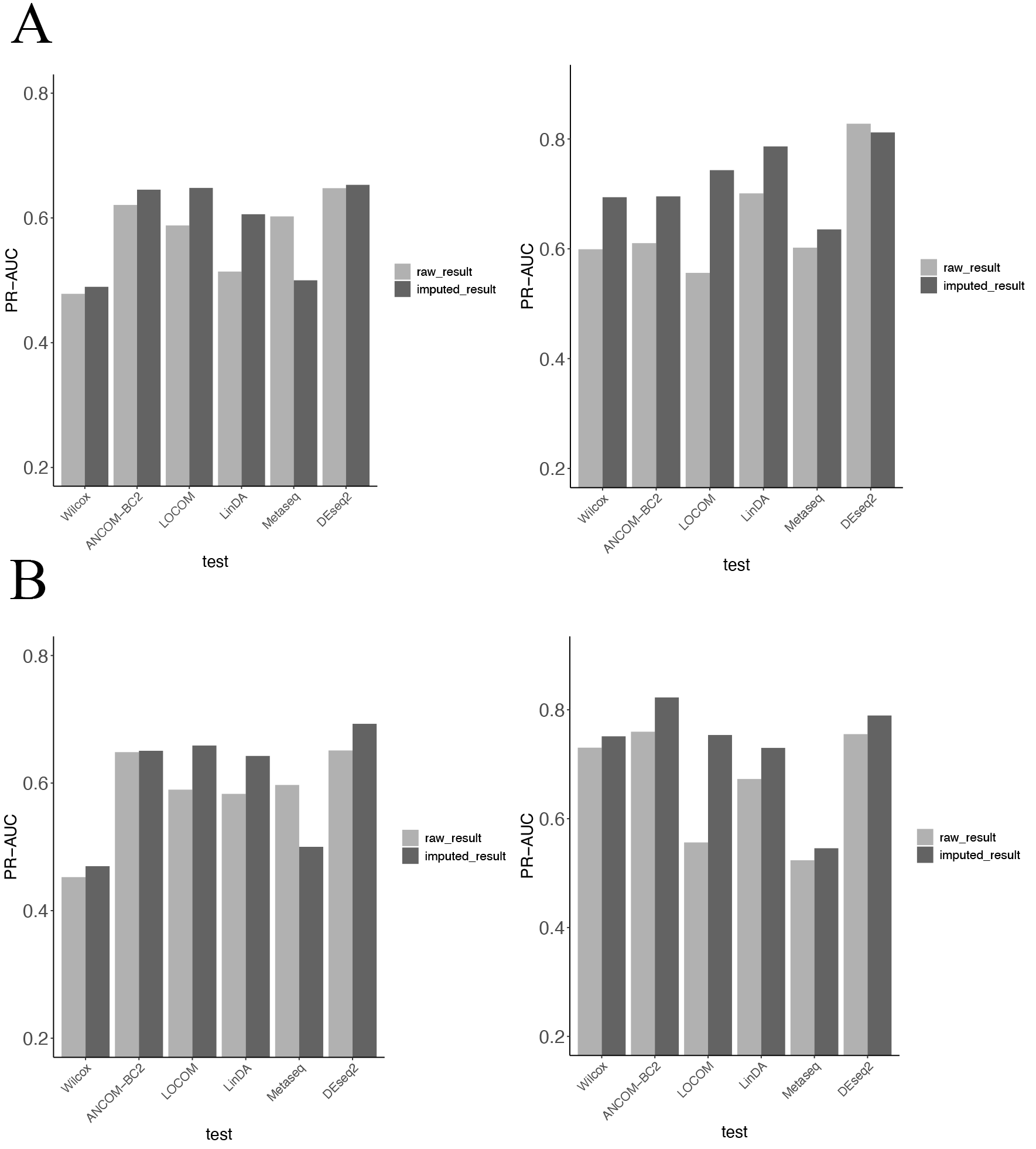

Supplement: S13 Fig — The left graph shows the predictive classification results for the Qin et al. [38] dataset using differentially abundant (DA) taxa as features obtained from Karlsson et al. [36]‘s original dataset (light-colored bars) and the dataset imputed by TphPMF (dark-colored bars); the right graph displays the predictive classification results for the Karlsson et al. [36] dataset using DA taxa as features obtained from Qin et al. [38]’s original dataset (light-colored bars) and the dataset imputed by TphPMF (dark-colored bars). A. Linear kernel Support Vector Machine (SVM). B. Gaussian kernel Support Vector Machine (SVM). (TIF) [file pcbi.1012858.s013.tif]
