# Supplementary material for: TphPMF: A microbiome data imputation method using hierarchical Bayesian Probabilistic Matrix Factorization
Source: PLoS Comput Biol. 2025 Mar 11;21(3):e1012858. doi: 10.1371/journal.pcbi.1012858 (PMC11957397; doi:10.1371/journal.pcbi.1012858)
Supplement: S1 Table — Here, h represents the phylogenetic hierarchy of the taxa, s denotes the samples (rows of the matrix), t denotes the taxa (columns of the matrix), p(m) represents the parent node, and c(m) represents the child node. (PDF) [file pcbi.1012858.s014.pdf]

S1 Table. Algorithm for iterative sampling in the Gibbs sampler. Here, h represents the phylogenetic hierarchy of the taxa, s denotes the samples (rows of the matrix), t denotes the taxa (columns of the matrix), p(m) represents the parent node, and c(m) represents the child node.

Algorithm S1 Gibbs Sampling for TphPMF

for $\mathrm{h} = 1, \cdots, \mathrm{H}$ **do**

  Initialize model parameters $\{S^{1(h)}, T^{1(h)}\}$

for $\mathrm{s} = 1, \cdots, \mathrm{S}$ **do**

  for $\mathrm{h} = \mathrm{H}, \cdots, 1$ **do** ⊳ bottom-up

    for each $\mathrm{m} = 1, \cdots, \mathrm{M}$ sample $\mathbf{t}_\mathrm{m}$ in parallel :

    $\mathbf{t}_\mathrm{m}^{s+1(h)} \sim p\left(\mathbf{t}_\mathrm{m}^{s(h)} \middle| \mathbf{y}_\mathrm{m}^{(h)}, S^{s(h)}, \mathbf{t}_{p(m)}^{s(h-1)}, \mathbf{t}_{c(m)}^{s(h+1)}\right)$

    for each $\mathrm{n} = 1, \cdots, \mathrm{N}$ sample $\mathbf{s}_\mathrm{n}$ in parallel:

    $\mathbf{s}_\mathrm{n}^{s+1(h)} \sim p\left(\mathbf{s}_\mathrm{n}^{s(h)} \middle| \mathbf{y}_\mathrm{n}^{(h)}, T^{s+1(h)}, \mathbf{s}_{n}^{s(h-1)}, \mathbf{s}_{n}^{s(h+1)}\right)$

  for $\mathrm{h} = 1, \cdots, \mathrm{H}$ **do** ⊳ top-down

    for each $\mathrm{m} = 1, \cdots, \mathrm{M}$ sample $\mathbf{t}_\mathrm{m}$ in parallel:

    $\mathbf{t}_\mathrm{m}^{s+2(h)} \sim p\left(\mathbf{t}_\mathrm{m}^{s+1(h)} \middle| \mathbf{y}_\mathrm{m}^{(h)}, S^{s+1(h)}, \mathbf{t}_{p(m)}^{s+1(h-1)}, \mathbf{t}_{c(m)}^{s+1(h+1)}\right)$

    for each $\mathrm{n} = 1, \cdots, \mathrm{N}$ sample $\mathbf{s}_\mathrm{n}$ in parallel:

    $\mathbf{s}_\mathrm{n}^{s+2(h)} \sim p\left(\mathbf{s}_\mathrm{n}^{s+1(h)} \middle| \mathbf{y}_\mathrm{n}^{(h)}, T^{s+2(h)}, \mathbf{s}_{n}^{s+1(h-1)}, \mathbf{s}_{n}^{s+1(h+1)}\right)$
