# Supplementary material for: TphPMF: A microbiome data imputation method using hierarchical Bayesian Probabilistic Matrix Factorization
Source: PLoS Comput Biol. 2025 Mar 11;21(3):e1012858. doi: 10.1371/journal.pcbi.1012858 (PMC11957397; doi:10.1371/journal.pcbi.1012858)
Supplement: S2 Table — (PDF) [file pcbi.1012858.s015.pdf]

S2 Table. Comparison of the MSE between the TphPMF-imputed and complete datasets across four key parameter variations in Simulation 1.

the height of cutting the clustering tree at the "S" level	the height of cutting the clustering tree at the "G" level	the height of cutting the clustering tree at the "F" level	the size of the latent vectors	MSE
5	10	15	1	**0.0113489**
2	10	15	1	**0.0116921**
8	10	15	1	**0.0353683**
5	8	15	1	**0.0511469**
5	12	15	1	**0.0397202**
5	10	13	1	**0.0356664**
5	10	17	1	**0.0437593**
5	10	15	1.5	**0.0352698**
5	10	15	2.5	**0.0121588**
5	10	15	5	**0.0213391**
