# Supplementary material for: TphPMF: A microbiome data imputation method using hierarchical Bayesian Probabilistic Matrix Factorization
Source: PLoS Comput Biol. 2025 Mar 11;21(3):e1012858. doi: 10.1371/journal.pcbi.1012858 (PMC11957397; doi:10.1371/journal.pcbi.1012858)
Supplement: S1 File — (DOCX) [file pcbi.1012858.s016.docx]

**TphPMF: a microbiome data imputation method using hierarchical Bayesian Probabilistic Matrix Factorization**

**Supplementary Materials**

**Simulation 1 for benchmarking imputation methods**

To compare TphPMF with several existing popular genomic-data estimation methods [1-6], we generated microbiome abundances from a generative model fitted to the T2D data with 53 subjects and 344 taxa (Karlsson et al., 2013 [7]). Below we describe the data generation process step by step.

**Complete data generation**

1. We followed the data pre-processing steps of TphPMF (see Materials and methods in the main text) to convert the OTU count matrix to log-transformed normalized abundances. Then we removed the taxa with greater than 95% of zero counts (equivalently, $\log_{10}\left( 1.01 \right)$ abundances) across subjects and kept 193 taxa. We denote the abundance matrix after this filtering step by $Y=\left( Y_{\mathrm{ij}} \right)\in R_{>0}^{N\times M}$, where $N=53$and $M=193$.
2. Based on the method used in TphPMF to identify the taxonomic group abundances that need to be estimated (see Materials and methods in the main text), we have defined a set of non-biological zero points, which is denoted as $\Omega$. The complement of this set, denoted as $\Omega^{c}$, contains taxonomic group abundances that are unlikely missing, and hence do not require estimation.
3. Under the assumption that

$$p\left( S | \sigma_{S}^{2} \right)=\prod_{i=1}^{N} \mathcal{N}\left( S_{i} | 0,\sigma_{S}^{2}I \right), p\left( T | \sigma_{T}^{2} \right)=\prod_{j=1}^{M} \mathcal{N}\left( T_{j} | 0,\sigma_{T}^{2}I \right)$$

we fitted the following model:

$$p\left( Y | S,T,\sigma^{2} \right)=\prod_{i=1}^{N} \prod_{j=1}^{M} \left[ \mathcal{N}\left( Y_{\mathrm{ij}} | S_{i}^{T}T_{j},\sigma^{2} \right) \right]^{I_{\mathrm{ij}}}$$

by minimizing the sum of squared errors objective function with a quadratic regularization term:

$$E=\frac{1}{2}\sum_{i=1}^{N} \sum_{j=1}^{M} I_{\mathrm{ij}}\left( Y_{\mathrm{ij}}-S_{i}^{T}T_{j} \right)^{2}+\frac{\lambda_{S}}{2}\sum_{i=1}^{N} \left\| S_{i} \right\|^{2}+\frac{\lambda_{T}}{2}\sum_{j=1}^{M} \left\| T_{j} \right\|^{2}$$

where $I_{\mathrm{ij}}=1$ when $Y_{\mathrm{ij}}\in\Omega^{c}$; otherwise, it equals 0. The row vectors $S_{i}$​and $T_{j}$represent the latent feature vectors for a specific sample and a specific taxonomic group, respectively. $\sigma_{S}^{2}, \sigma_{T}^{2}$ and$\sigma^{2}$ are the variances of the observational noise and are hyperparameters. $\lambda_{T}={\sigma^{2}}/{\sigma_{T}^{2}}$ and $\lambda_{S}={\sigma^{2}}/{\sigma_{S}^{2}}$. Then we obtained the parameter estimates: $\hat{S_{i}}, \hat{T_{j}}$ and $\hat{\sigma}$.

1. We generated the complete, log-transformed abundance of taxon j in sample i : ${Y_{\mathrm{ij}}}^{\mathrm{comp}}$ by randomly sampling from the normal distribution : $\mathcal{N}\left( {\hat{S_{i}}}^{T}\hat{T_{j}},\hat{\sigma}^{2} \right)$. We denote the resulting matrix $Y^{\mathrm{comp}}=\left( {Y_{\mathrm{ij}}}^{\mathrm{comp}} \right)$as the complete data that contain log-transformed abundances without missing values.

**Zero-inflated data generation**

Next, we introduced zero inflation to $Y^{\mathrm{comp}}$and generated $Y^{\mathrm{zi}}$ by mimicking real data as follows. With the identified $\Omega^{c}$, we calculated $z_{k}^{\mathrm{real}}$(taxon k’s proportion of likely false zeros across samples) and $u_{k}^{\mathrm{real}}$ (taxon k’s average abundance after we excluded likely false zeros) for each taxon k in Karlsson et al.’s [7] data Y, $k=1,\cdots,M$. Next we introduced zeros into $Y^{\mathrm{comp}}$ in the following non-parametric way for each taxon j, $j=1,\cdots,M$ :

1. We calculated taxon j’s average abundance in $Y^{\mathrm{comp}}$ as the mean of column j, denoted by $u_{j}^{\mathrm{comp}}$;
2. We randomly sampled a value from $\left\{ z_{k}^{\mathrm{real}}:u_{k}^{\mathrm{real}}\in\left( u_{j}^{\mathrm{comp}}-0.5,u_{j}^{\mathrm{comp}}+0.5 \right) \right\}$ and denoted it as $z_{j}^{\mathrm{zi}}$, i.e., the proportion of false zeros to be introduced into taxon j’s abundances in $Y^{\mathrm{comp}}$;
3. We randomly drew false zero indicators $I_{\mathrm{ij}}\sim Bernoulli\left( z_{j}^{\mathrm{zi}} \right)$ independently for sample $i=1,\cdots,N$ ;
4. We set $Y_{\mathrm{ij}}^{\mathrm{zi}}=max\left( {Y_{\mathrm{ij}}}^{\mathrm{comp}}\cdot I_{\mathrm{ij}}, \log_{10}\left( 1.01 \right) \right)$ .

**Evaluation criteria for imputation accuracy**

After applying an imputation method to $Y^{\mathrm{zi}}$, we obtained $Y^{\mathrm{imp}}$ and evaluated the imputation performance by the following three criteria. Note that TphPMF identified 8 taxa with too many zeros in $Y^{\mathrm{zi}}$ and excluded them from imputation. For a fair comparison, we excluded these 8 taxa from the calculation of the evaluation criteria for every imputation method, so M was reduced to $193-8=185$ in the following.

1. Mean squared error (MSE) between $Y^{\mathrm{imp}}$ and $Y^{\mathrm{comp}}$:

$$MSE=\frac{1}{\mathrm{NM}}\sum_{i=1}^{N} \sum_{j=1}^{M} \left( {Y_{\mathrm{ij}}}^{\mathrm{comp}}-{Y_{\mathrm{ij}}}^{\mathrm{imp}} \right)^{2},$$

which is shown in Fig 1A.

1. Pearson correlation between ${Y_{\cdot j}}^{\mathrm{imp}}$ and ${Y_{\cdot j}}^{\mathrm{comp}}$ for $j=1,\cdots,M$. The mean of these M correlations is shown in Fig 1B.
2. Mean and standard deviation (SD) of taxon j in the imputed data vs. those in the complete data:

$\bar{Y}_{\cdot j}^{\mathrm{imp}}=\frac{1}{N}\sum_{i=1}^{N} {Y_{\mathrm{ij}}}^{\mathrm{imp}}$ vs. $\bar{Y}_{\cdot j}^{\mathrm{comp}}=\frac{1}{N}\sum_{i=1}^{N} {Y_{\mathrm{ij}}}^{\mathrm{comp}} ,$

$${\mathrm{sd}_{\cdot j}}^{\mathrm{imp}}=\sqrt{\frac{1}{N-1}\sum_{i=1}^{N} \left( {Y_{\mathrm{ij}}}^{\mathrm{imp}}-\bar{Y}_{\cdot j}^{\mathrm{imp}} \right)^{2}}$$

vs.

$${\mathrm{sd}_{\cdot j}}^{\mathrm{comp}}=\sqrt{\frac{1}{N-1}\sum_{i=1}^{N} \left( {Y_{\mathrm{ij}}}^{\mathrm{comp}}-\bar{Y}_{\cdot j}^{\mathrm{comp}} \right)^{2}},$$

$j=1,\cdots,M$.

In Fig 1C, we computed the Wasserstein distance between the distribution of $\left\{ {\bar{Y}_{\cdot1}^{\mathrm{imp}}}/{{\mathrm{sd}_{\cdot1}}^{\mathrm{imp}}},\cdots,{\bar{Y}_{\cdot M}^{\mathrm{imp}}}/{{\mathrm{sd}_{\cdot M}}^{\mathrm{imp}}} \right\}$

vs. that of

$\left\{ {\bar{Y}_{\cdot1}^{\mathrm{comp}}}/{{\mathrm{sd}_{\cdot1}}^{\mathrm{comp}}},\cdots,{\bar{Y}_{\cdot M}^{\mathrm{comp}}}/{{\mathrm{sd}_{\cdot M}}^{\mathrm{comp}}} \right\}$.

**Simulation 2 for benchmarking imputation methods**

Similar to the simulation 1, this simulation was also based on the whole-genome sequencing (WGS) dataset of Type 2 Diabetes (T2D) samples collected by Karlsson et al. in 2013[7].

**Complete data generation**

1. We followed the data pre-processing steps of TphPMF (see Materials and methods in the main text) to convert the OTU count matrix to log-transformed normalized abundances. Then we removed the taxa with greater than 95% of zero counts (equivalently, $\log_{10}\left( 1.01 \right)$abundances) across subjects and kept 193 taxa. We denote the abundance matrix after this filtering step by $Y=\left( Y_{\mathrm{ij}} \right)\in R_{>0}^{N\times M}$, where $N=53$and $M=193$.
2. Based on the method used in TphPMF to identify the taxonomic group abundances that need to be estimated (see Materials and methods in the main text), we have defined a set of non-biological zero points, which is denoted as $\Omega$. The complement of this set, denoted as $\Omega^{c}$, contains taxonomic group abundances that are unlikely missing, and hence do not require estimation.
3. Then we fitted a linear model that combines information of similar samples and similar taxonomic groups in the count matrix to the (sample, taxon) pairs in $\Omega^{c}$ [3]:

$$Y_{\mathrm{ij}}=Y_{i\cdot}^{T}\alpha_{j}+Y_{\cdot j}^{T}\beta_{i}+\epsilon_{\mathrm{ij}}$$

by minimizing the loss function

$$\sum_{\left( i,j \right)\epsilon\Omega^{c}} \left( Y_{\mathrm{ij}}-\left( Y_{i\cdot}^{T}\alpha_{j}+Y_{\cdot j}^{T}\beta_{i} \right) \right)^{2}+\lambda\left( \sum_{j=1}^{M} \sum_{j^{'}\neq j}^{M} D_{jj^{'}}^{\psi}\left| \alpha_{jj^{'}} \right|+\sum_{i=1}^{N} \sum_{i^{'}\neq i}^{N} \left| \beta_{ii^{'}} \right| \right)$$

where $Y_{i\cdot}\in R_{>0}^{M}$ represents the abundance of M taxonomic groups in the i-th sample, $\alpha_{j}$ represents the weights of M taxonomic groups for predicting the abundance of the j-th taxonomic group (the j-th term being zero); $Y_{\cdot j}\in R_{>0}^{N}$ represents the abundance of taxonomic group j across N samples, $\beta_{i}$ represents the weights for N samples when predicting sample i (the i-th term being zero); $\epsilon_{\mathrm{ij}}$is the error term. The tuning parameters $\lambda, \psi\geq0$ were chosen by cross-validation. Note that we constrained $\alpha_{j}$ to have only 5 non-zero entries corresponding to the 5 taxa closest to taxon j in phylogenetic distance, i.e.,

$\left\{ j^{'}:D_{jj^{'}} is among the five smallest of all D_{\mathrm{jr}},r\neq j \right\}$.

Then we obtained the parameter estimates: $\hat{\alpha_{j}}\in R^{M}, \hat{\beta_{i}}\in R^{N},i=1,\cdots,N;j=1,\cdots,M$.

1. We generated the complete, log-transformed abundance of taxon j in sample i, as

${Y_{\mathrm{ij}}}^{\mathrm{comp}}=Y_{i\cdot}^{T}\hat{\alpha_{j}}+Y_{\cdot j}^{T}\hat{\beta_{i}}$.

We denote the resulting matrix $Y^{\mathrm{comp}}=\left( {Y_{\mathrm{ij}}}^{\mathrm{comp}} \right)$as the complete data that contain log-transformed abundances without missing values.

**Zero-inflated data generation**

We introduced zero inflation to $Y^{\mathrm{comp}}$ and generated $Y^{\mathrm{zi}}$ , following the same non-parametric procedure as in simulation 1.

**Evaluation criteria for imputation accuracy**

This section is also similar to that of simulation 1.

**Simulation 3 for benchmarking imputation methods based on real WGS data**

To further benchmark TphPMF against several widely used genomic-data estimation methods, we employed a semi-simulation approach by obtaining a subset of microbiome WGS data without missing values from a T2D dataset composed of 96 subjects (53 T2D and 43 control subjects) and 344 taxa (Karlsson et al., 2013)[7]. The data generation process is described below.

**Complete data generation**

1. We followed the data pre-processing steps of TphPMF (see Materials and methods in the main text) to convert the OTU count matrix to log-transformed normalized abundances.
2. Based on the method used in TphPMF to identify the taxonomic group abundances that need to be estimated (see Materials and methods in the main text), we have defined a set of non-biological zero points, which is denoted as $\Omega$. The complement of this set, denoted as $\Omega^{c}$, contains taxonomic group abundances that are unlikely missing, and hence do not require estimation.
3. We removed the taxa with greater than 85% of zero counts (equivalently, $\log_{10}\left( 1.01 \right)$abundances) across subjects and kept 146 taxa.
4. For each subject, we checked if it has at least 73 non-zero counts (equivalently, greater than $\log_{10}\left( 1.01 \right)$), across the 146 taxa. If yes, we kept the subject; otherwise, we filtered it out. This step left us with 60 subjects. And we denote the filtered abundance matrix by $Y=\left( Y_{\mathrm{ij}} \right)\in R_{>0}^{N\times M}$, where $N=60$and $M=146$.
5. For the abundance matrix $Y$, we define the set $A=\left\{ Y_{\mathrm{ij}}>\log_{10}\left( 1.01 \right),i=1,\cdots,60;j=1,\cdots,146 \right\}$ as the set of non-zero values. We then replaced each $Y_{\mathrm{ij}}\in A^{c}$(i.e., the missing or zero values) for $i=1,\cdots,60;j=1,\cdots,146$ with normally distributed random numbers, $N_{\mathrm{ij}}\sim\mathcal{N}\left( \mu,\sigma^{2} \right)$, where $\mu$ is the mean and $\sigma$ is the standard deviation of the abundance values in set $A$.
6. We generated the complete, log-transformed abundance of taxon j in sample i, as

$${Y_{\mathrm{ij}}}^{\mathrm{comp}}=\left\{ \begin{matrix} Y_{\mathrm{ij}} , \mathrm{if}Y_{\mathrm{ij}}\in A. \\ N_{\mathrm{ij}} , \mathrm{if}Y_{\mathrm{ij}}\in A^{c}. \end{matrix} \right.$$

We denote the resulting matrix $Y^{\mathrm{comp}}=\left( {Y_{\mathrm{ij}}}^{\mathrm{comp}} \right)$as the complete data that contain log-transformed abundances without missing values.

**Zero-inflated data generation**

Next, we introduced zero inflation to $Y^{\mathrm{comp}}$and generated $Y^{\mathrm{zi}}$ by mimicking real data as follows. With the identified $\Omega^{c}$, we calculated $z_{k}^{\mathrm{real}}$(taxon k’s proportion of likely false zeros across samples) and $u_{k}^{\mathrm{real}}$ (taxon k’s average abundance after we excluded likely false zeros) for each taxon k in Karlsson et al.’s [7] data Y, $k=1,\cdots,M$. Next we introduced zeros into $Y^{\mathrm{comp}}$ in the following non-parametric way for each taxon j, $j=1,\cdots,M$ :

1. We calculated taxon j’s average abundance in $Y^{\mathrm{comp}}$ as the mean of column j, denoted by $u_{j}^{\mathrm{comp}}$;
2. We randomly sampled a value from $\left\{ z_{k}^{\mathrm{real}}:u_{k}^{\mathrm{real}}\in\left( u_{j}^{\mathrm{comp}}-0.5,u_{j}^{\mathrm{comp}}+0.5 \right) \right\}$ and denoted it as $z_{j}^{\mathrm{zi}}$, i.e., the proportion of false zeros to be introduced into taxon j’s abundances in $Y^{\mathrm{comp}}$;
3. We randomly drew false zero indicators $I_{\mathrm{ij}}\sim Bernoulli\left( z_{j}^{\mathrm{zi}} \right)$ independently for sample $i=1,\cdots,N$ ;
4. We set $Y_{\mathrm{ij}}^{\mathrm{zi}}=max\left( {Y_{\mathrm{ij}}}^{\mathrm{comp}}\cdot I_{\mathrm{ij}}, \log_{10}\left( 1.01 \right) \right)$

**Evaluation criteria for imputation accuracy**

After applying an imputation method to $Y^{\mathrm{zi}}$, we obtained $Y^{\mathrm{imp}}$ and evaluated the imputation performance by the following two criteria.

1. Mean squared error (MSE) between $Y^{\mathrm{imp}}$ and $Y^{\mathrm{comp}}$:

$MSE=\frac{1}{\mathrm{NM}}\sum_{i=1}^{N} \sum_{j=1}^{M} \left( {Y_{\mathrm{ij}}}^{\mathrm{comp}}-{Y_{\mathrm{ij}}}^{\mathrm{imp}} \right)^{2}$.

1. Pearson correlation between ${Y_{\cdot j}}^{\mathrm{imp}}$ and ${Y_{\cdot j}}^{\mathrm{comp}}$ for $j=1,\cdots,M$. The mean of these M correlations is shown in S1 Fig.

**References**

1. Hastie T, Mazumder R. softimpute: Matrix completion via iterative soft-thresholded svd. R package version. 2015;1(1).

2. Huang M, Wang J, Torre E, Dueck H, Shaffer S, Bonasio R, et al. SAVER: gene expression recovery for single-cell RNA sequencing. Nature methods. 2018;15(7):539-42.

3. Jiang R, Li WV, Li JJ. mbImpute: an accurate and robust imputation method for microbiome data. Genome biology. 2021;22(1):192.

4. Li WV, Li JJ. An accurate and robust imputation method scImpute for single-cell RNA-seq data. Nature communications. 2018;9(1):997.

5. Linderman GC, Zhao J, Kluger Y. Zero-preserving imputation of scRNA-seq data using low-rank approximation. BioRxiv. 2018:397588.

6. Zeng Y, Li J, Wei C, Zhao H, Wang T. mbDenoise: microbiome data denoising using zero-inflated probabilistic principal components analysis. Genome Biology. 2022;23(1):94.

7. Karlsson FH, Tremaroli V, Nookaew I, Bergström G, Behre CJ, Fagerberg B, et al. Gut metagenome in European women with normal, impaired and diabetic glucose control. Nature. 2013;498(7452):99-103.
